# Supplementary material for: Chiral evasion and stereospecific antifolate resistance in Staphylococcus aureus
Source: PLoS Comput Biol. 2022 Feb 10;18(2):e1009855. doi: 10.1371/journal.pcbi.1009855 (PMC8865654; doi:10.1371/journal.pcbi.1009855)
Supplement: S1 Text — Contains a list of acronyms used in this manuscript, crystallographic data collection and refinement statistics, descriptions of and data on biochemical experiments for NADPH, supplementary structure figures, and a discussion of the normalization and conversion between K* scores and IC50. Table A. List of acronyms used in this manuscript. Table B. Crystallographic structure data collection and refinement statistics for 7T7Q and 7T7S. Table C. Equilibrium ratios of α/β-NADPH and isolated yields of t-NADPH under various conditions. Fig A. 1H NMR shows acid-mediated transformation of β-NADPH to t-NADPH. Fig B. Diastereomers observed via 1H NMR from the cyclization reaction of NADPH. Fig C. 1H NMR shows thermal isomerization at 100°C. Fig D. HPLC traces of β-NADPH, α-NADPH, and t-NADPH. Fig E. Pre-incubation with t-NADPH increases the inhibition potency of R-27. Fig F. Minor populations in low-energy ensemble of t-NADPH binary complexes. (PDF) [file pcbi.1009855.s001.pdf]

Supplementary Text: Chiral evasion and stereospecific antifolate resistance in *Staphylococcus aureus*

Siyu Wang[1,2¶], Stephanie M. Reeve[3¶], Graham T. Holt[1,2¶], Adegoke A. Ojewole[1,2¶¤], Marcel S. Frenkel[4], Pablo Gainza[1], Santosh Keshipeddy[3], Vance G. Fowler[5], Dennis L. Wright[3,6], Bruce R. Donald[1,4,7,8*],

1 Department of Computer Science, Duke University, Durham, North Carolina, USA
2 Program in Computational Biology and Bioinformatics, Duke University, Durham, North Carolina, USA
3 Department of Pharmaceutical Sciences, University of Connecticut, Storrs, Connecticut, USA
4 Department of Biochemistry, Duke University Medical Center, Durham, North Carolina, USA
5 Division of Infections Diseases, Department of Medicine, Duke University Medical Center, Durham, North Carolina, USA
6 Department of Chemistry, University of Connecticut, Storrs, Connecticut, USA
7 Department of Mathematics, Duke University, Durham, North Carolina, USA
8 Department of Chemistry, Duke University, Durham, North Carolina, USA

¶These authors contributed equally to this work.
¤Current Address: Genentech, Inc., San Francisco, California, USA
*Corresponding Author: brd+pcb21@cs.duke.edu

Table A: Summary of acronyms used in this work.

Acronyms	Full names
DHFR	dihydrofolate reductase
SaDHFR	*Staphylococcus aureus* dihydrofolate reductase
TMP	trimethoprim
PLA	propargyl-linked antifolate
NADPH	nicotinamide adenine dinucleotide phosphate
MRSA	methicillin-resistant *Staphylococcus aureus*
SSTI	skin and soft tissue infection
DHF	dihydrofolate
THF	tetrahydrofolate
PABA	para-aminobenzoic acid
CPD	computational protein design
t-NADPH	tricyclic NAPDH
OSPREY	Open-Source Protein REdesign for You (a structure-based computational protein design software suite developed by our lab)
IC_{50}	half maximal inhibitory concentration
K_a	binding constant
PF	partition function
PDB	the Protein Data Bank archive

Table B: Crystallographic structure data collection and refinement statistics for 7T7Q and 7T7S.

	SaDHFR:R-27:α-NADPH	SaDHFR:R-27:t-NADPH
PDB ID	7T7Q	7T7S
Space group	$P6_122$	$P6_122$
No. monomers in asymmetric unit	1	1
Unit cell (a, b, c in Å)	78.88, 78.88, 107.9<br>90, 90, 120	78.88, 78.88, 107.9<br>90, 90, 120
Resolution (Å)	28.86-2.20	28.86-2.20
Completeness % (last shell %)	97.9 (84.0)	97.9 (84.0)
Unique reflections	10393	10393
$\langle I/\sigma \rangle$ (last shell)	80.81 (1.62)	80.81 (1.62)
R_{work}/R_{free}	0.1841/0.2543	0.1840/0.2529
No. of atoms (protein, ligands, solvent)	1406 (1280, 80, 46)	1406 (1280, 80, 46)
Rms deviation bond lengths (Å), angles (deg)	0.008, 0.89	0.008, 0.89
Average B factor for protein (Å^2)	37.06	37.10
Average B factor for ligand (Å^2)	36.22	35.99
Average B factor for solvent molecules (Å^2)	37.68	37.66
Residues in most favored regions (%)	97.42	97.42
Residues in additional allowed regions (%)	1.94	1.94
Residues in disallowed regions (%)	0.65	0.65
Collection location	BNL NSLS X25	BNL NSLS X25

Biochemical experiments for α-NADPH and t-NADPH

Analysis of NADPH populations and isolation of t-NADPH

We describe convenient synthetic methods to access pure t-NADPH and enriched α-NADPH isomers by rapid equilibration of commercially available β-NADPH at different pH and temperatures. After thermal isomerization the α-NADPH fraction was isolated in (≈70:30) along with the β-isomer, which is beneficial for biochemical experiments. Interestingly, we have found that under physiological conditions (37 °C) the same α-NADPH fraction leads directly to the cyclic form (c-THN)-TPN or t-NADPH.

Attempts to synthesize t-NADPH using previously reported conditions for anomerization of NADH (*8*) led to rapid degradation of the cofactor. We therefore screened a variety of mild acid and buffer conditions and found that malic acid at pH 4.5 cleanly yielded the desired t-NADPH in a reasonable 30% yield after purification. The product slowly

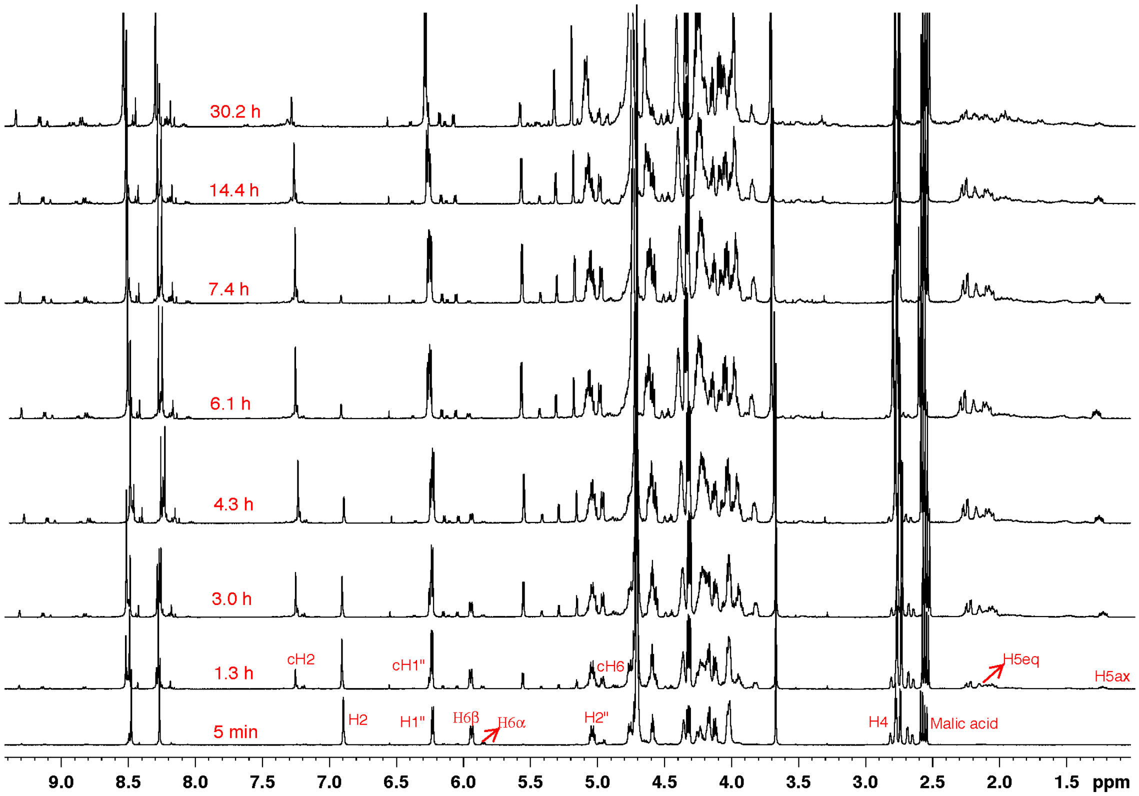

Figure A: ^{1}H NMR shows acid-mediated transformation of β-NADPH to t-NADPH.

decomposed to ADP and nicotinamide overtime if continuously exposed to acidic conditions. This type of decomposition was previously observed with cyclic-a-NADH also named as (c-THN)AD (α-O2'-6B-cyclotetrahydronicotinamide adenine dinucleotide) in acid medium. (*7*) Therefore, careful reaction monitoring by NMR was performed to determine the optimal time point at which there is a maximum accumulation of t-NADPH. ^{1}H NMRs were collected every 30 min and we have observed that after 7 h there is maximum product formation with minor amounts of decomposition products observed (Figure A).

During the study, we observed the transient formation of the α-NADPH intermediate in an 86:14 (β:α NADPH) equilibrium ratio. The presence of α-NADPH was confirmed by analogy to the NADH β:α ratio (90:10) and assignment of the H6 proton of (α-NADPH) peak at 5.84 ppm (*9*). Two possible configurations result from the cyclization reaction yielding two diastereomers in an 85:15 ratio, confirmed by ^{1}H NMR. Formation of the major diastereomer can be explained by the attack of the 2'-hydroxyl group to the nicotinamide ring to give the more stable product. From the coupling constants of C6H

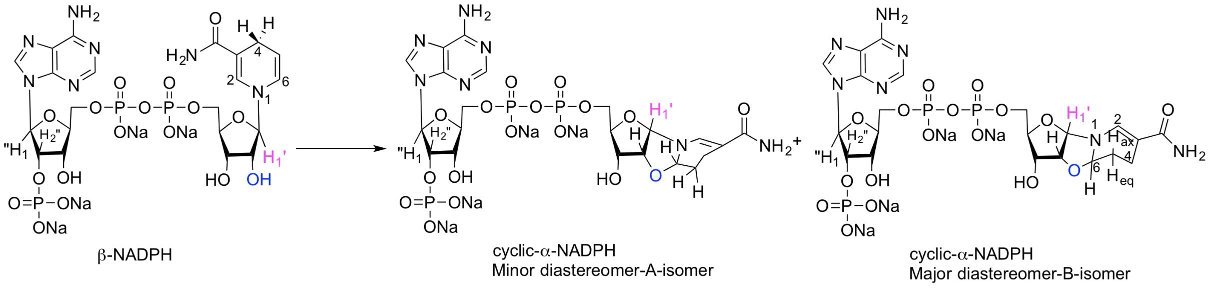

Figure B: Diastereomers observed via ^{1}H NMR from the cyclization reaction.

to C5H_{ax} and C5H_{eq} (9.5 and 4.2 Hz) the major diastereomer was assigned by analogy to t-NADPH (Figure B) (*10*).

As the α-NADPH is an intermediate leading to the stable t-NADPH under acidic conditions, thermal isomerization experiments were evaluated as a potential preparation of the non-cyclic isomer (*9*). Excitingly, isomerization of β-NADPH to α-NADPH was achieved in D_2O at 100°C (Figure C). After 10 min, the equilibrium ratio was similar to that reached in the acid mediated reaction and the reaction was quenched by adjusting the reaction mixture to pH 8 with saturated ammonium carbonate (Table C)

Later, α-NADPH was isolated using HPLC to give an enriched fraction of α-NADPH (≈70:30) along with the beta-isomer (Figure Dii). If allowed to stand for up to 18 h at room temperature the fraction reached ≈50:50 distribution, allowing reasonable window for using the enriched mixture in biological experiments. t-NADPH was not observed at room temperature during equilibration. Interestingly, however when the same fraction was incubated at 37°C overnight t-NADPH was observed (Figure Dii). Also, t-NADPH was found to be stable in D_2O at pH 7 for 1 week.

NMR and HPLC methods

All reactions were conducted under an atmosphere of Argon in 5 mL glass vial. 1H NMR analysis was performed in a reaction NMR tube in D_2O. Ammonium carbonate (≥30 ammonia basis), β-NADPH tetrasodium salt, malic acid and other chemicals used in this study were purchased from commercial sources. ^{1}H NMR spectra were recorded at 400 MHz, and/or at 500 MHz and calibrated to the D_2O peak at 4.70 ppm. ^{13}C NMR spectra were recorded at 100MHz, and/or at 125 MHz. HSQC spectra were recorded at 500 and/or 600 MHz. Chemical shifts are reported in units of parts per million (ppm). High-resolution mass spectra were obtained on an AccuTOF instrument using ESI technique. Reaction products were purified using HPLC on a C18 column (250 mm x 4.6 mm) with particle size 5 mm, mobile phase: 100% 0.01M ammonium carbonate or 0.01 M tris buffer with flow rate of 0.8 mL/min.

α-O2′-6B-cyclo-1,4,5,6-tetrahydronicotinamide adenine dinucleotide phosphate (1). To β-NADPH (25 mg) dissolved in water (1.1 mL) was added 89 μL of 0.71 M aqueous

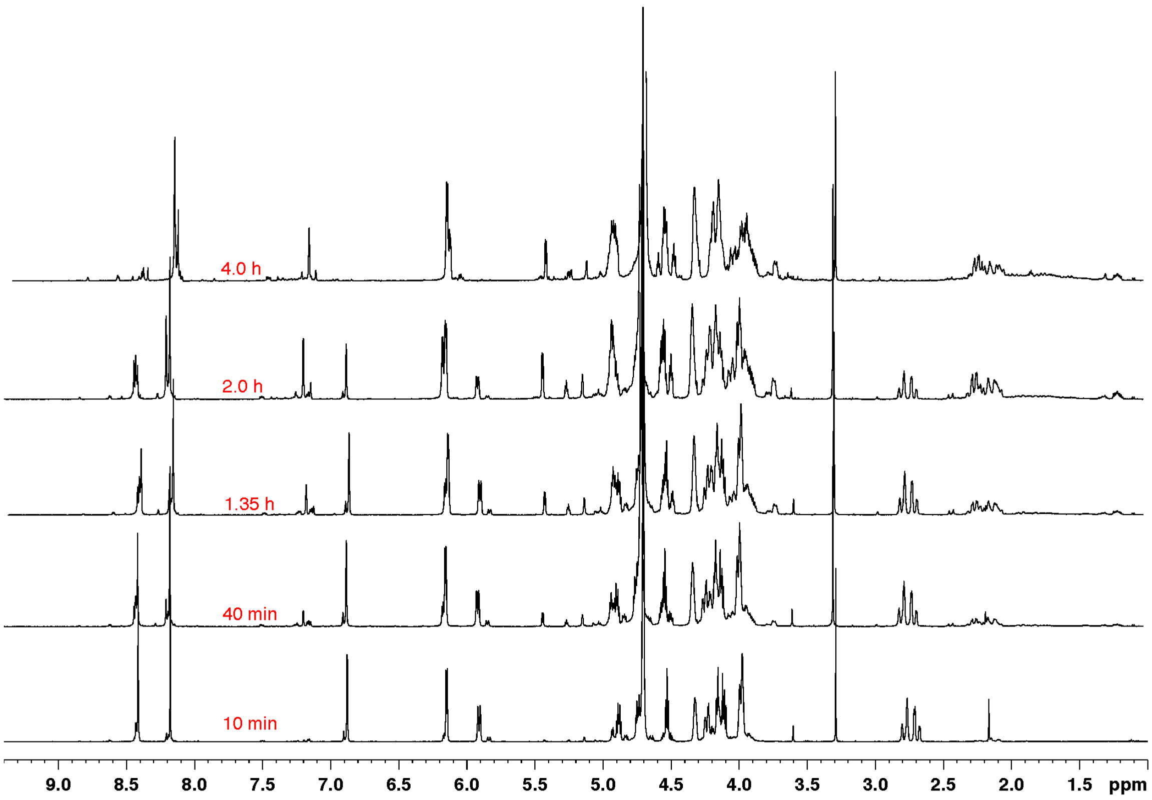

Figure C: ^{1}H NMR shows thermal isomerization at 100°C.

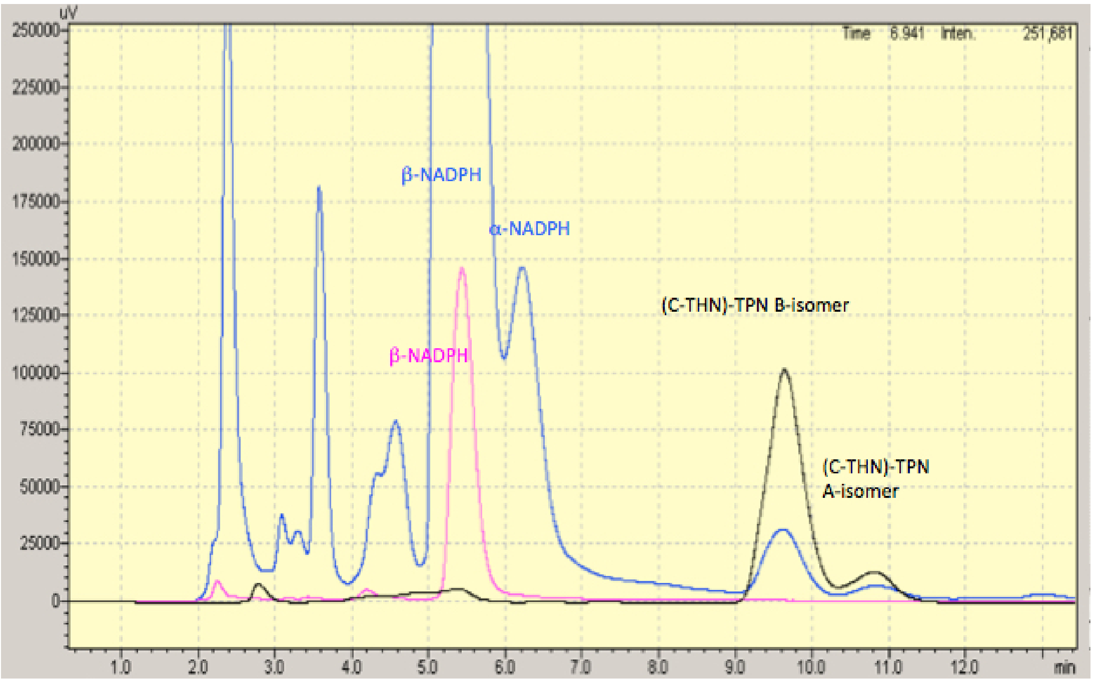

(i) Blue trace: thermal isomerization reaction at 100°C after 10 min. Pink trace: commercially-available β-NADPH. Black trace: Pure t-NADPH control.

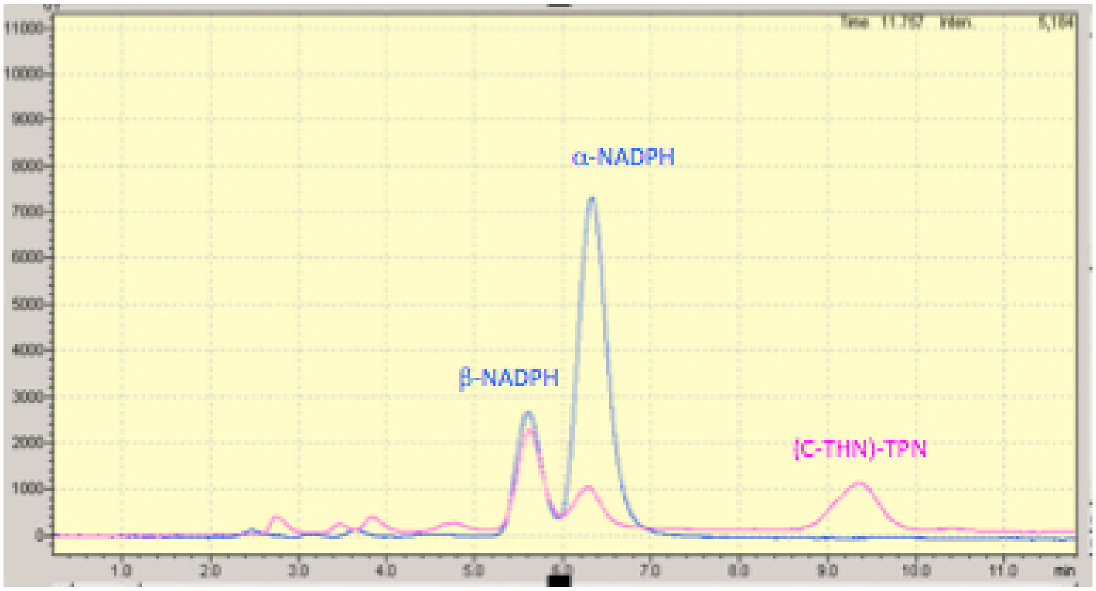

(ii) Blue trace: enriched α-NADPH fraction. Pink trace: Incubation of enriched α-NADPH at 37°C overnight.

Figure D: HPLC traces of β-NADPH, α-NADPH, and t-NADPH.

solution of malic acid and stirred at rt for 7 h. Reaction was quenched by adjusting the pH to 8.0 with saturated ammonium carbonate. Solvent was evaporated and the crude mixture was redissolved in minimum amount of water (1 mL) and purified using HPLC to yield the required t-NADPH (1) as white solid (7.5 mg, 30% yield). Yield of the compound was determined using proton NMR by adding pyridine as a control; 1H NMR (500 MHz, D_2O) δ 8.45 (s, 1H), 8.18 (s, 1H), 7.19 (s, 1H), 6.14 (d, J = 3.7 Hz, 1H), 5.43 (d, J = 4.4 Hz, 1H), 4.92 (m, 1H), 4.92 (dd, J = 9.5, 4.0, 1H), 4.53 (dd, J = 4.7, 4.7 Hz, 1H), 4.48 (dd, J = 4.7, 4.7 Hz, 1H), 4.32 (m, 1H), 4.18 (m, 2H), 4.04 (m, 1H), 3.98 (dd, J = 5.6, 5.6 Hz, 1H), 3.89 (m, 1H), 3.73 (m, 1H), 2.25 (ddd, J = 16.1, 5.0, 2.1 Hz, 1H), 2.16 (m, 1H), 2.08 (m, 1H), 1.20 (dddd, J = 14.4, 12.1, 9.4, 5.3 Hz, 1H); 13C NMR (125 MHz, D_2O) (major and minor diastereomers) δ 172.8, 155.6, 152.8, 149.1, 140.3, 137.3, 135.8, 118.6, 101.5, 101.2, 91.7, 89.3, 88.4, 87.9, 86.7 (d, J_{C-P} = 8.4 Hz), 82.9 (d, J_{C-P} = 8.6 Hz), 80.8, 80.4 (d, J_{C-P} = 8.1 Hz), 79.7, 76.3 (d, J_{C-P} = 3.7 Hz), 70.2, 70.0, 69.9, 65.2 (d, J_{C-P} = 4.2 Hz), 64.3 (d, J_{C-P} = 4.7 Hz), 63.4, 26.3, 25.8,18.9, 18.3; 31P NMR (500 MHz, D_2O) δ 3.37, -11.38, -11.38; HRMS (ESI, $[M\text{-}4Na+2H]^{-2}$) m/z 371.5406 (calculated for $C_{21}H_{28}N_7O_{17}P_3$, 743.0755; $[M\text{-}4Na+2H]^{-2}$ 371.5377)

t-NADPH is not a substrate of DHFR

To assess whether or not t-NADPH is a substrate of DHFR 2 μg/mL SaDHFR was incubated with 0.1 mM DHF and with or without 0.1 mM of t-NADPH. After 5 minutes, 0.1 mM β-NADPH was added to the reaction and rates were compared. No appreciable consumption of DHF was observed before addition of β-NADPH, indicating that t-NADPH is not a substrate of SaDHFR.

Determination of IC_{50} for t-NADPH and R-27

Enzyme kinetics were performed in triplicate by monitoring the rate of NADPH oxidation by DHFR via absorbance at 340 nm. The reaction was performed at room temperature in buffer containing 20 mM TES, 50 mM KCl, 0.5 mM EDTA, 10 mM beta-mercaptoethanol and 1 mg/mL BSA. t-NADPH inhibition was determined by co-incubating 2 μg/mL DHFR with 0.1mM β-NADPH and varying concentrations of t-NADPH and the enzyme reaction was activated by adding 0.1M DHF. The IC_{50} of t-NADPH is defined as the concentration of t-NADPH that was required to inhibit the enzyme to 50% of its activity without inhibitor.

IC_{50} values for R-27 were determined with and without co-incubation with t-NADPH. This assay follows the standard enzyme kinetic protocol above, with the modification in which the enzyme reaction is activated with a mix of 0.1mM β-NADPH and 0.1mM NADPH and the IC_{50} of R-27 is determined with 5 minute incubation of DHFR, R-27 and with or without 40 μM purified t-NADPH. See Figure E.

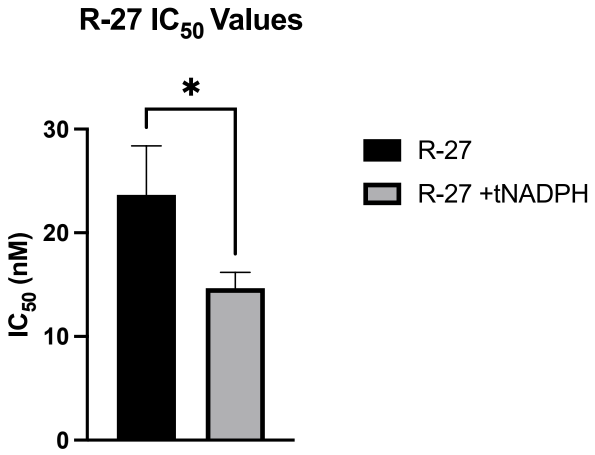

Figure E: **Pre-incubation with t-NADPH increases the inhibition potency of R-27.** SaDHFR was incubated with R-27 and with or without 40 μM of t-NADPH. The enzyme reaction was activated with a mixture of β-NADPH and DHF following previously-published conditions (*4*), see Section . Experiments performed in triplicate, error bars denote standard deviation, $p = 0.0349$.

Table C: **Equilibrium ratios of α/β-NADPH and isolated yields of t-NADPH under various conditions.** An observable population of α-NADPH is present at all tested conditions. Significant amounts of t-NADPH can be isolated in most cases. [1]Identified by ^{1}H NMR. [2]Observed by HPLC. [3]Incubation of an enriched α-NADPH fraction at 37 °C for 10 h. See Section .

Temp (°C)	pH	β:α NADPH ratio[1]	t-NADPH isolated yield
100	6.1	88.12	15% in 2 h
37	7.0	93:7	trace[2] in 12 h
25	4.5	86:14	30%[3] in 7 h
25	7.0	98.5:1.5	0%

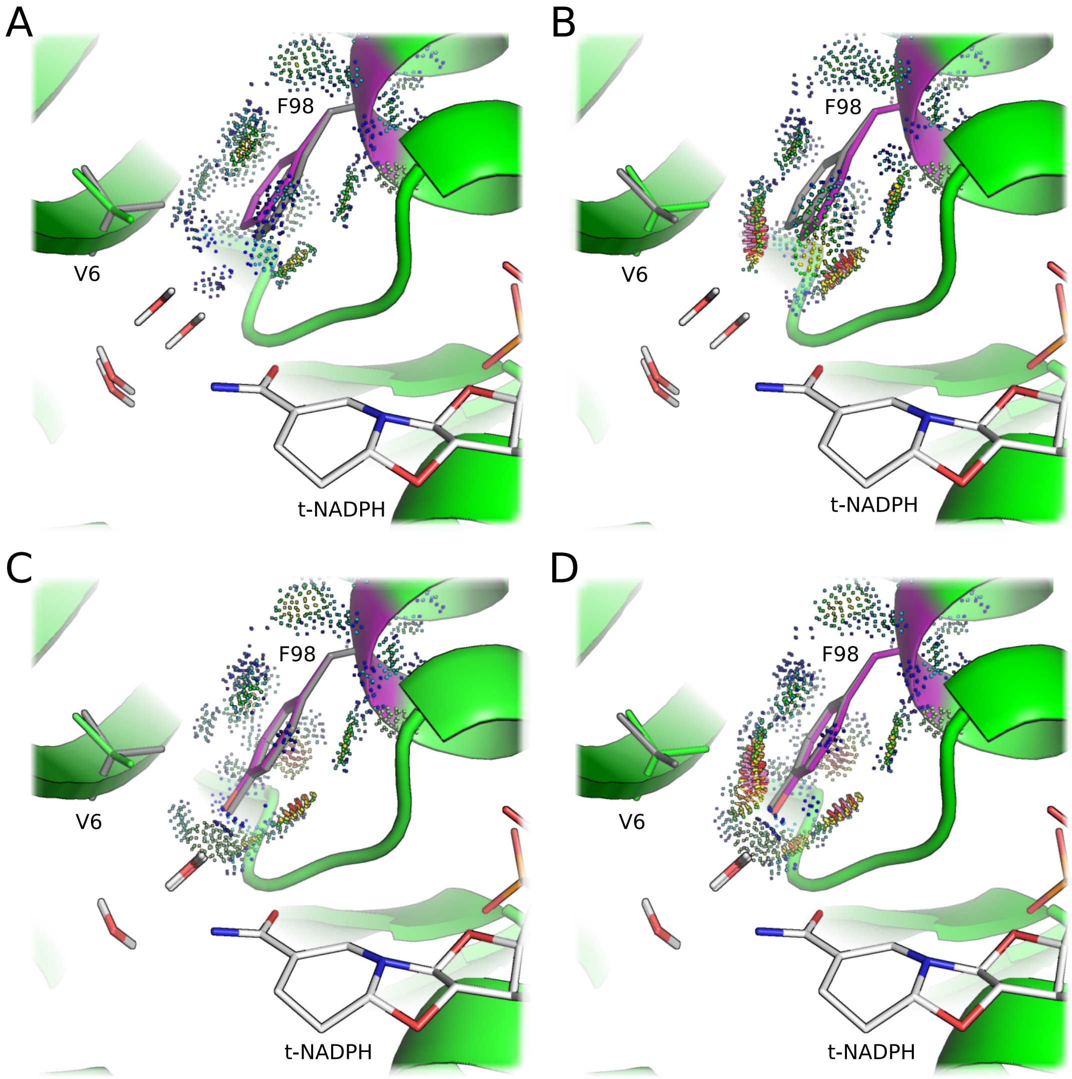

Figure F: **Minor populations in low-energy ensemble of t-NADPH binary complexes.** SaDHFR shown in green, with the critical residue 98 (F or Y) shown in purple and gray and t-NADPH shown in white. Atomic interactions of the relevant population of residue 98 (indicated in purple) shown using Probe dots (*13*), where green and blue dots indicate favorable interactions and red and purple lines indicate more unfavorable interactions. Both the SaDHFR:t-NADPH binary complex (A, B) and the SaDHFR(F98Y):t-NADPH binary complex (C,D), show minor populations for residues 98 and V6 in the low-energy ensemble. Major populations (A,C) are charactized by favorable interactions, but less-favorable minor populations (B,D) may indicate space in the active site relative to ternary or β-NADPH binary complexes. Notably only a single population of residues 98 and V6 is predicted for the low-energy ensembles for all ternary complexes and binary complexes with β-NADPH. For all ternary complexes, and for the SaDHFR(F98Y):β-NADPH binary complex, the single population is characterized by unfavorable interactions (See the main manuscript).

Normalization of K* scores

K* scores have been demonstrated many times to be successful in predicting the ranking of K_a for various mutants of given biological systems (*6, 11, 12*). However, sometimes the correlation between K* scores and K_a is not observed to be strictly quantitative (*1, 11*). A few reasons are known to explain such phenomenon: first, our current K* computations only focused on modeling flexibility of resides near the active sites; water molecules out of the binding pocket were not modeled explicitly, but were instead modeled by implicit model (EEF1 model (*5*)); and most physical effective energy functions are based on small-molecule energetics, which can overestimate van der Waals terms. The above limitations in the input model may result in overestimation or underestimation in energy and entropy. In order to compensate for these factors, acquire a better quantitative correlation between K* scores and experimental data and thereby make more accurate predictions, we sometimes normalize K* scores, i.e., scale K* scores of a given system by a constant factor (*2, 3, 6*). In our case here, we can observe from Table 3 that the lowest $\log_{10}$ K* score observed among all of our SaDHFR systems is 32.22 (R27:β-NADPH:DHFR(F98Y)). Since K_a must be greater than or equal to zero, we will assume that the zero point of K_a corresponds to $\log_{10}$ K* score approximately equal to 32 here in these cases. Therefore, we calculated a set of normalized $\log_{10}$ K* scores, which equals to the original $\log_{10}$ K* scores after subtracting an offset value of 32. Note that here we present K* scores in $\log_{10}$ space, and a subtraction by constant value in $\log_{10}$ space is equivalent to a multiplication in real space.

References

1. Mark A Hallen, Jeffrey W Martin, Adegoke Ojewole, Jonathan D Jou, Anna U Lowegard, Marcel S Frenkel, Pablo Gainza, Hunter M Nisonoff, Aditya Mukund, Siyu Wang, et al. OSPREY 3.0: Open-source protein redesign for you, with powerful new features. *Journal of computational chemistry*, 39(30):2494–2507, 2018.

2. Graham T Holt, Jonathan D Jou, Nicholas P Gill, Anna U Lowegard, Jeffrey W Martin, Dean R Madden, and Bruce R Donald. Computational analysis of energy landscapes reveals dynamic features that contribute to binding of inhibitors to CFTR-associated ligand. *The Journal of Physical Chemistry B*, 123(49):10441–10455, 2019.

3. Jonathan D Jou, Graham T Holt, Anna U Lowegard, and Bruce R Donald. Minimization-aware recursive K*: A novel, provable algorithm that accelerates ensemble-based protein design and provably approximates the energy landscape. *Journal of Computational Biology*, 27(4):550–564, 2020.

4. Santosh Keshipeddy, Stephanie M Reeve, Amy C Anderson, and Dennis L Wright. Nonracemic antifolates stereoselectively recruit alternate cofactors and overcome resistance in S. aureus. *Journal of the American Chemical Society*, 137(28):8983–8990, 2015.

5. Themis Lazaridis and Martin Karplus. Discrimination of the native from misfolded protein models with an energy function including implicit solvation. *Journal of molecular biology*, 288(3):477–487, 1999.

6. Anna U Lowegard, Marcel S Frenkel, Graham T Holt, Jonathan D Jou, Adegoke A Ojewole, and Bruce R Donald. Novel, provable algorithms for efficient ensemble-based computational protein design and their application to the redesign of the c-Raf-RBD: KRas protein-protein interface. *PLOS Computational Biology*, 16(6):e1007447, 2020.

7. Joy R. Miksic and Phyllis R. Brown. Reactions of Reduced Nicotinamide Adenine Dinucleotide in Acid: Studies by Reversed-Phase High-Pressure Liquid Chromatography. *Biochemistry*, 17(11):2234–2238, 1978.

8. Norman J Oppenheimer. The primary acid product of DPNH. *Biochemical and Biophysical Research Communications*, 50(3):683–690, 1973.

9. Norman J. Oppenheimer, Lyle J. Arnold, and Nathan O. Kaplan. A Structure of Pyridine Nucleotides in Solution. *Proceedings of the National Academy of Sciences*, 68(12):3200–3205, dec 1971.

10. Norman J Oppenheimer and Nathan O Kaplan. Structure of the primary acid rearrangement product of reduced nicotinamide adenine dinucleotide (NADH). *Biochemistry*, 13(23):4675–4685, 1974.

11. Stephanie M Reeve, Pablo Gainza, Kathleen M Frey, Ivelin Georgiev, Bruce R Donald, and Amy C Anderson. Protein design algorithms predict viable resistance to an experimental antifolate. *Proceedings of the National Academy of Sciences*, 112(3):749–754, 2015.

12. Kyle E Roberts, Patrick R Cushing, Prisca Boisguerin, Dean R Madden, and Bruce R Donald. Computational design of a PDZ domain peptide inhibitor that rescues CFTR activity. *PLoS Comput Biol*, 8(4):e1002477, 2012.

13. J Michael Word, Brent K Presley, Simon C Lovell, David C Richardson, and Robert C Batemanm. Exploring steric constraints on protein mutations using MAGE/PROBE. *Protein Science*, 9(11):2251–2259, 2000.
