# Supplementary figures and images for: Chiral evasion and stereospecific antifolate resistance in Staphylococcus aureus
Source: PLoS Comput Biol. 2022 Feb 10;18(2):e1009855. doi: 10.1371/journal.pcbi.1009855 (PMC8865654; doi:10.1371/journal.pcbi.1009855)

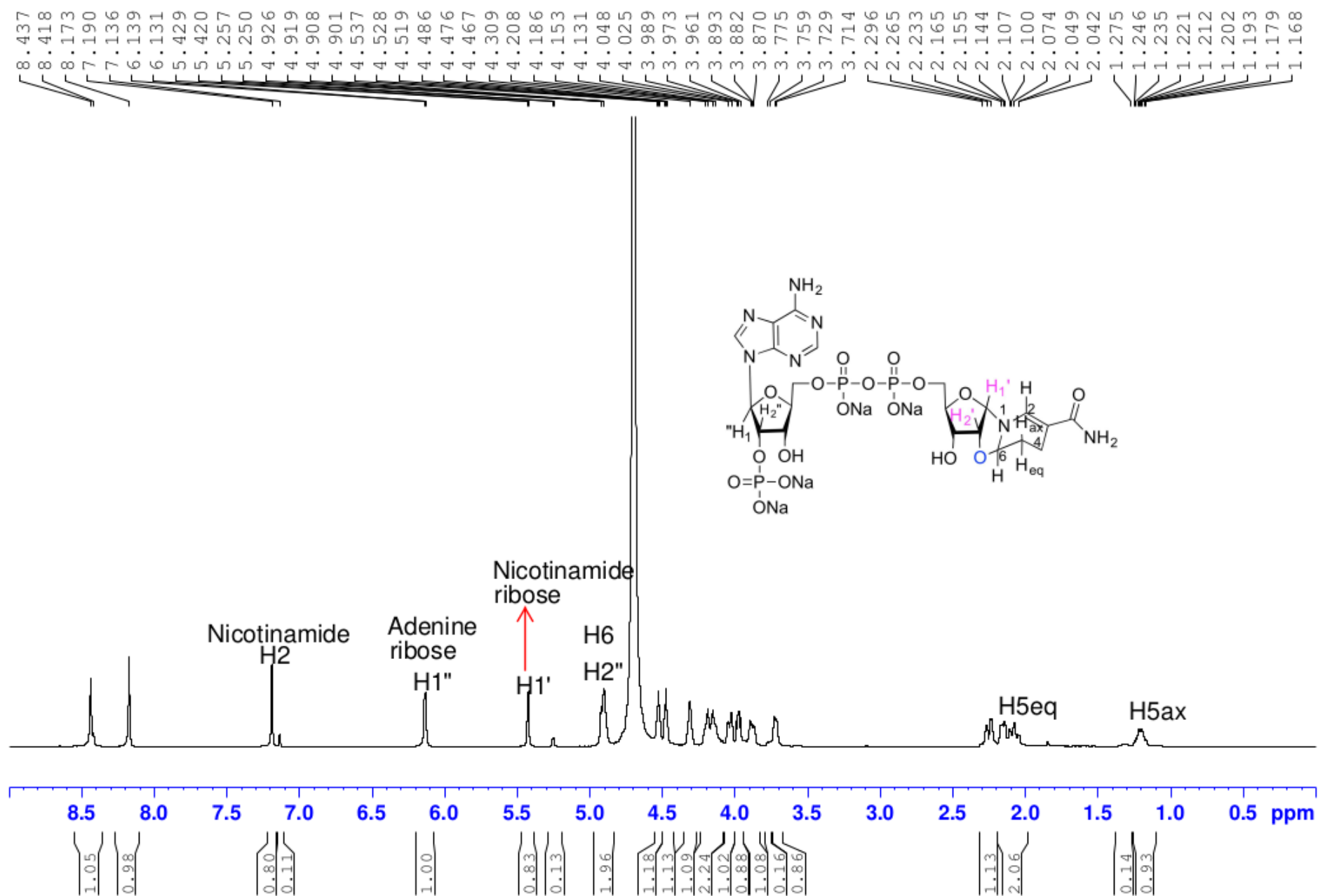

Supplement: S1 Data — (PDF) [file pcbi.1009855.s002.pdf]

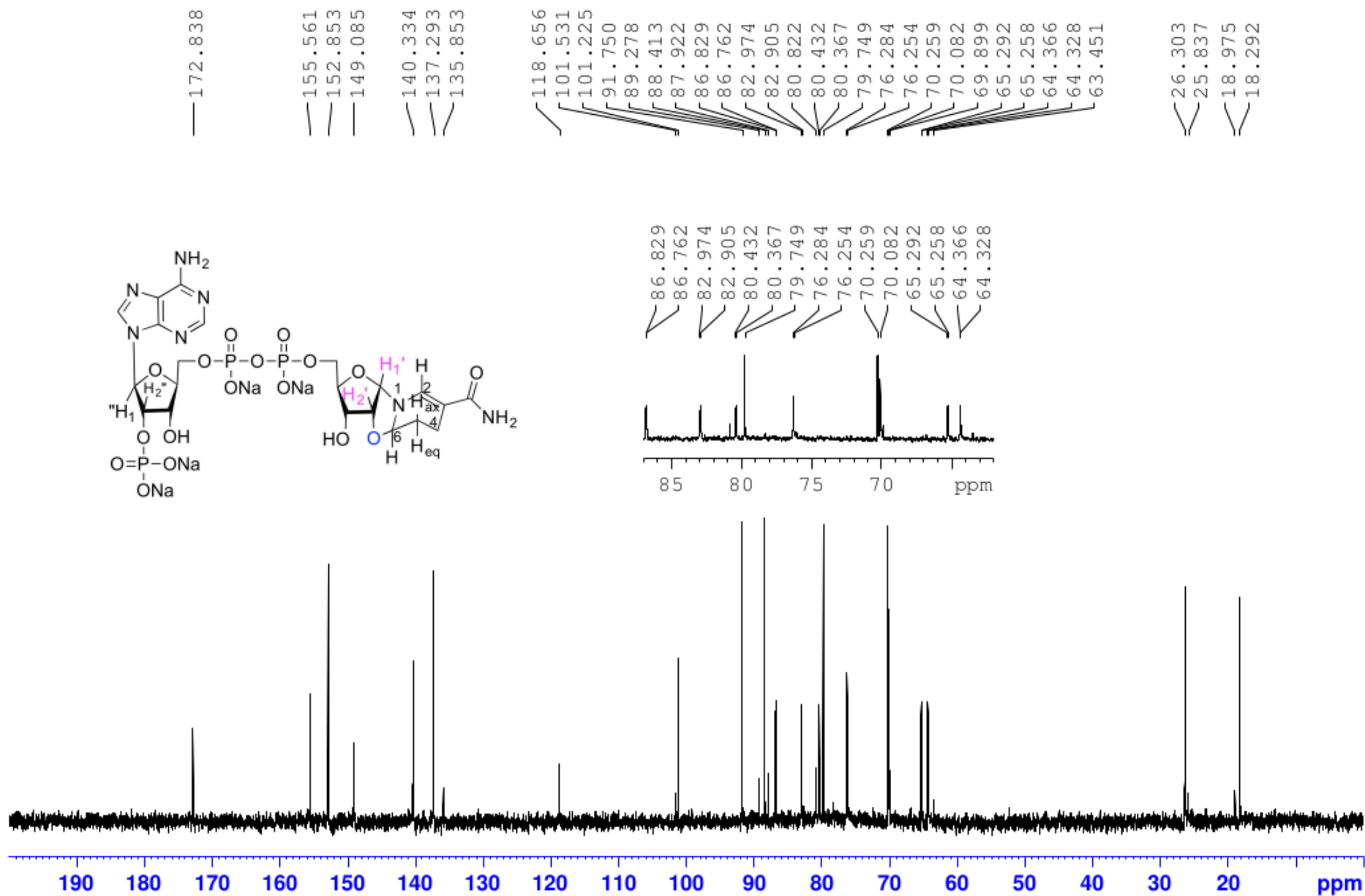

Supplement: S2 Data — (PDF) [file pcbi.1009855.s003.pdf]

"skk-nb5-cnadph purification with ammo.carbonate" 2 1 "C:\Users\santosh\Desktop\cnadph files"

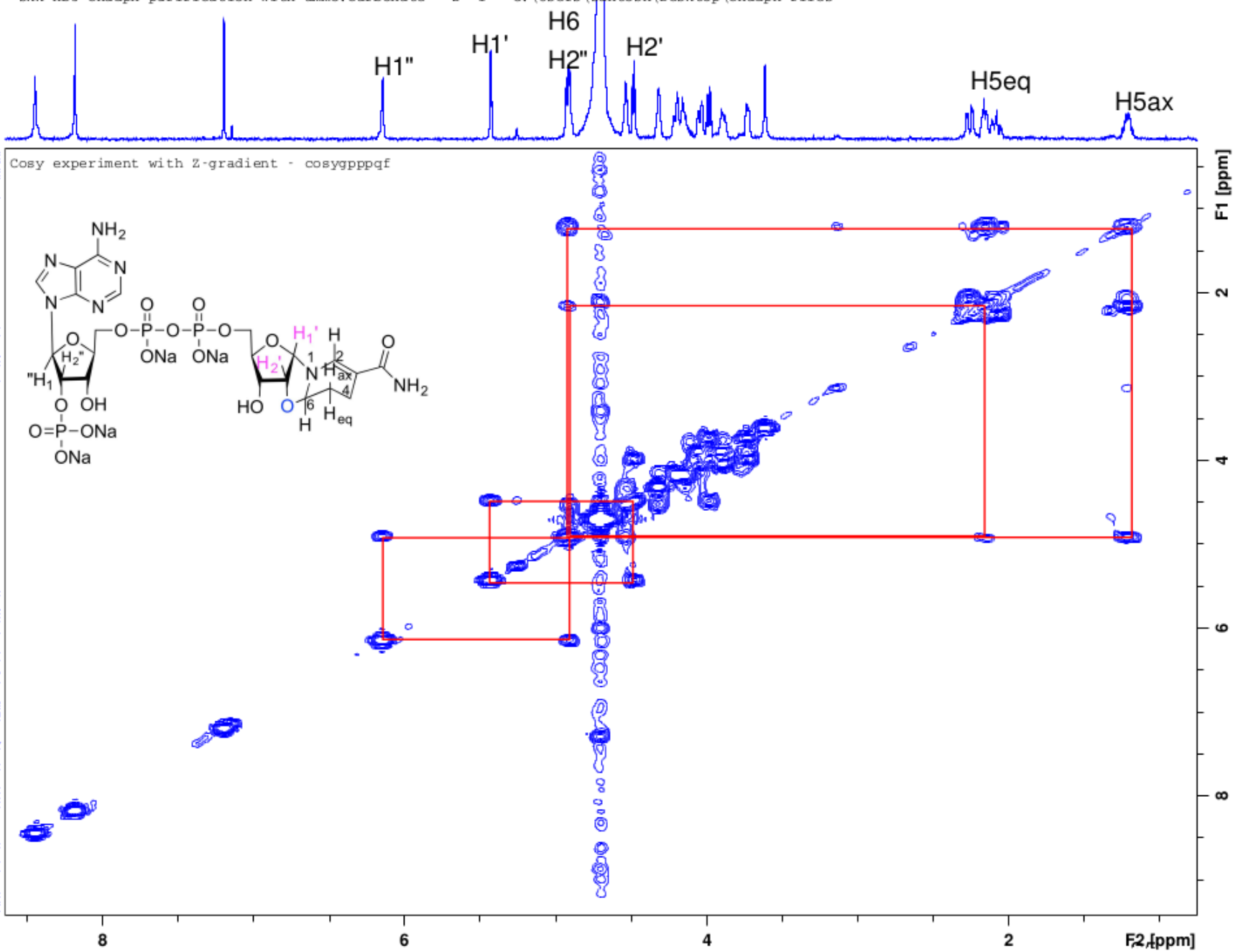

Supplement: S3 Data — (PDF) [file pcbi.1009855.s004.pdf]

"skk-nb5-cnadph purification with ammo.carbonate" 6 1 "C:\Users\santosh\Desktop\cnadph files"

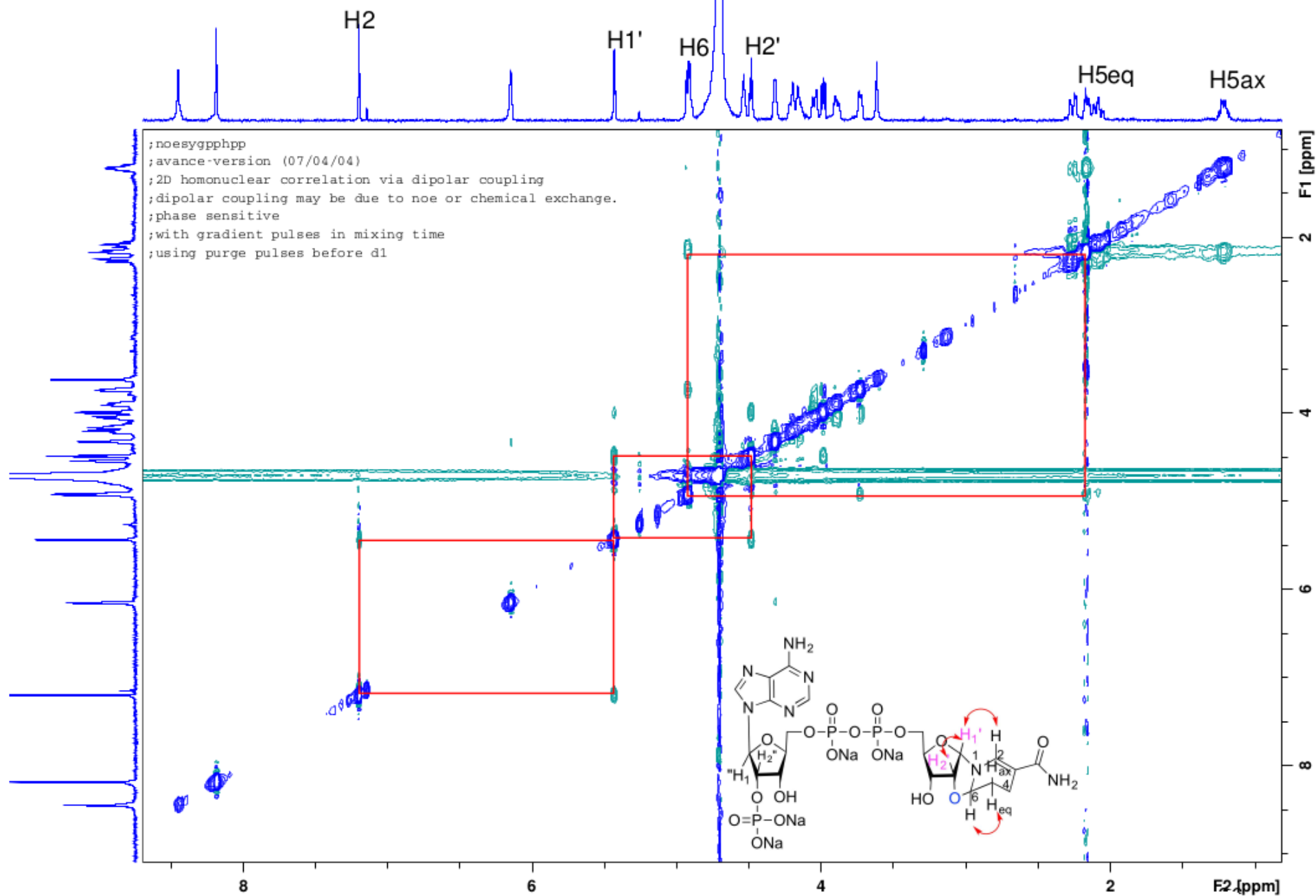

Supplement: S4 Data — (PDF) [file pcbi.1009855.s005.pdf]

HSQCAD\_20160613.fid 30 1 "C:\Users\nmrlab\Desktop\cnadph files"

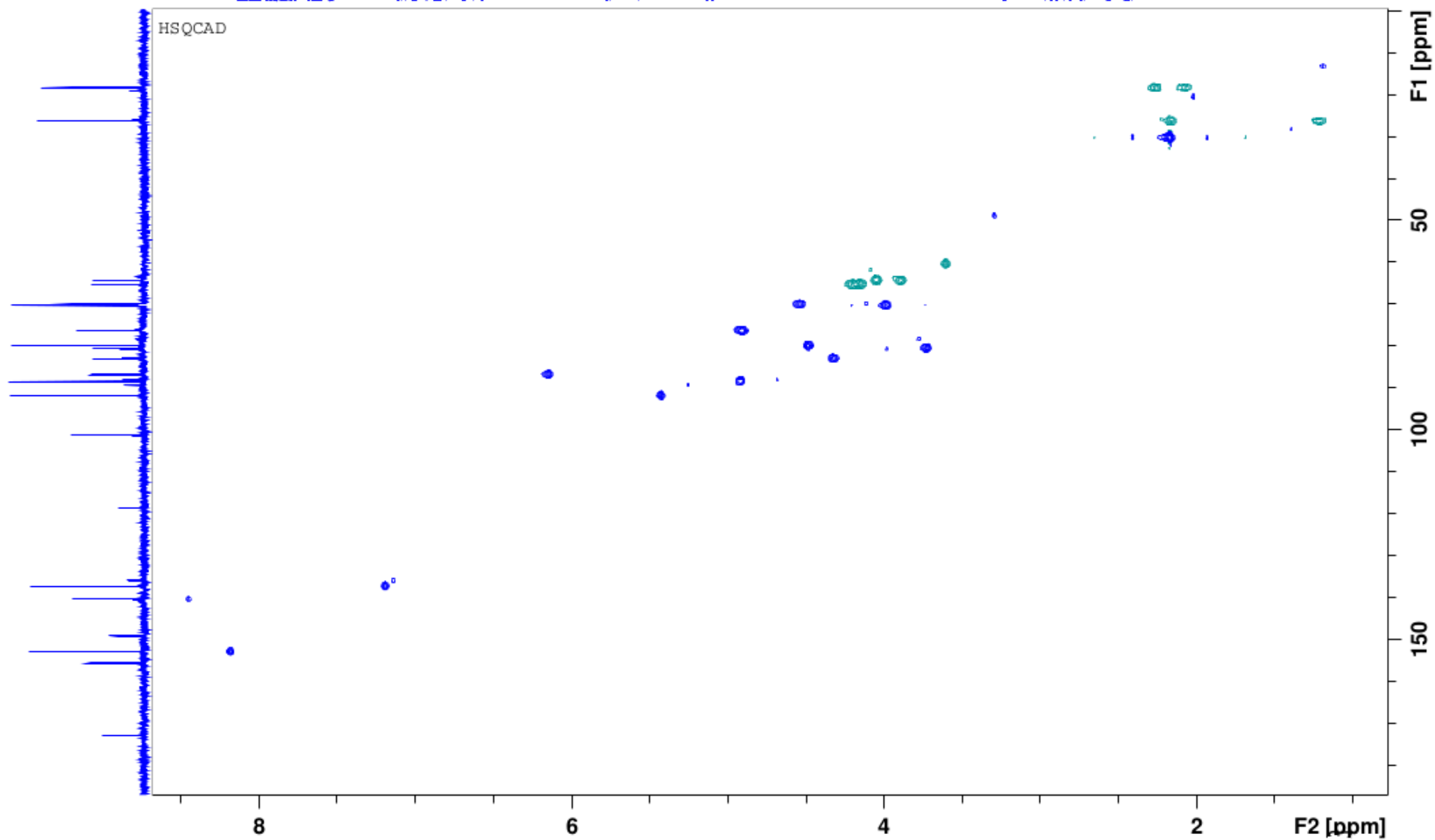

HSQCAD\_20160613.fid 30 1 "C:\Users\nmrlab\Desktop\cnadph files"

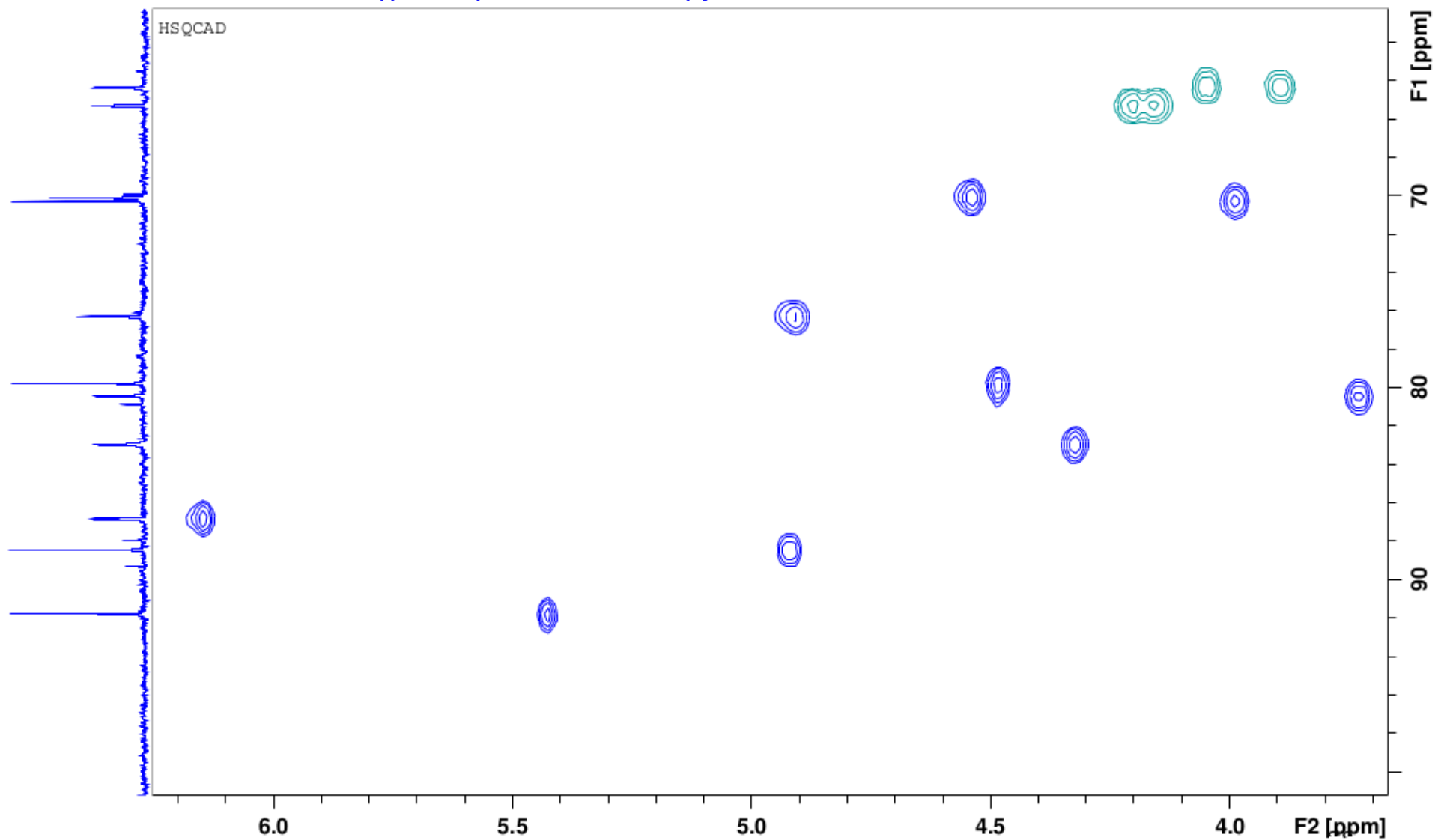

Supplement: S5 Data — (PDF) [file pcbi.1009855.s006.pdf]

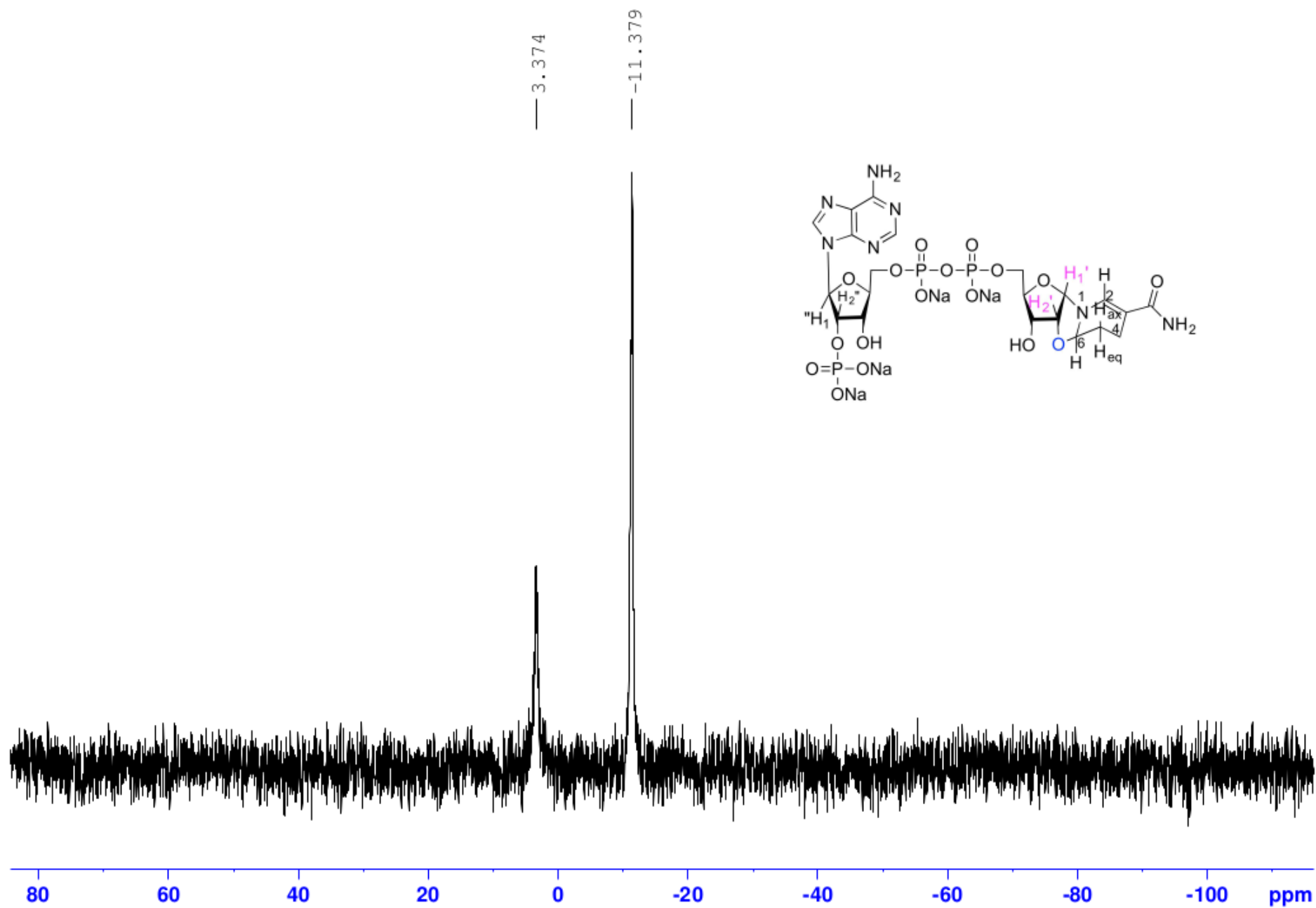

Supplement: S6 Data — (PDF) [file pcbi.1009855.s007.pdf]

<sup>1</sup>H spectrum

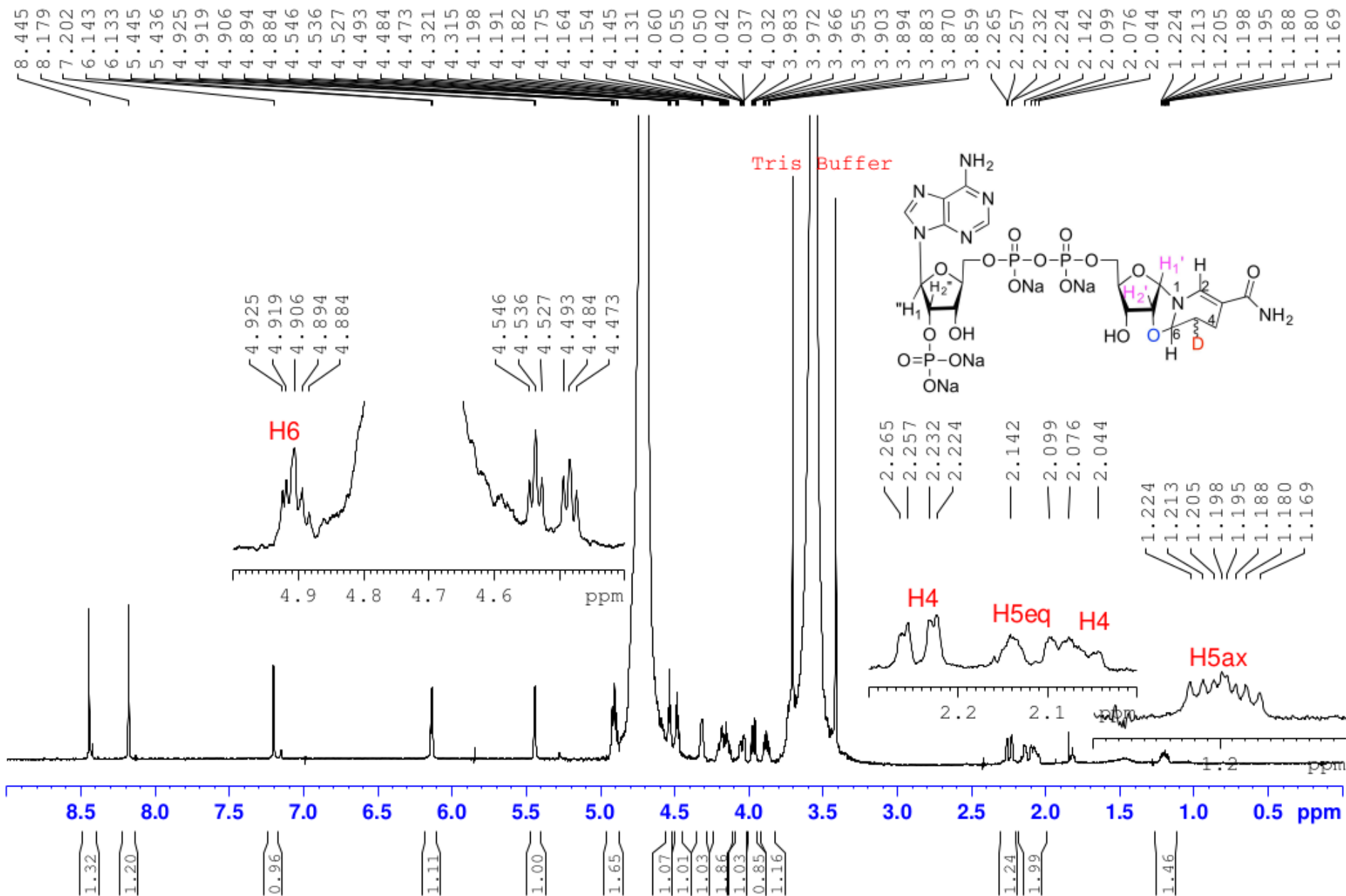

Supplement: S7 Data — (PDF) [file pcbi.1009855.s008.pdf]
