# Supplementary material for: Chiral evasion and stereospecific antifolate resistance in Staphylococcus aureus
Source: PLoS Comput Biol. 2022 Feb 10;18(2):e1009855. doi: 10.1371/journal.pcbi.1009855 (PMC8865654; doi:10.1371/journal.pcbi.1009855)
Supplement: S8 Data — (PDF) [file pcbi.1009855.s009.pdf]

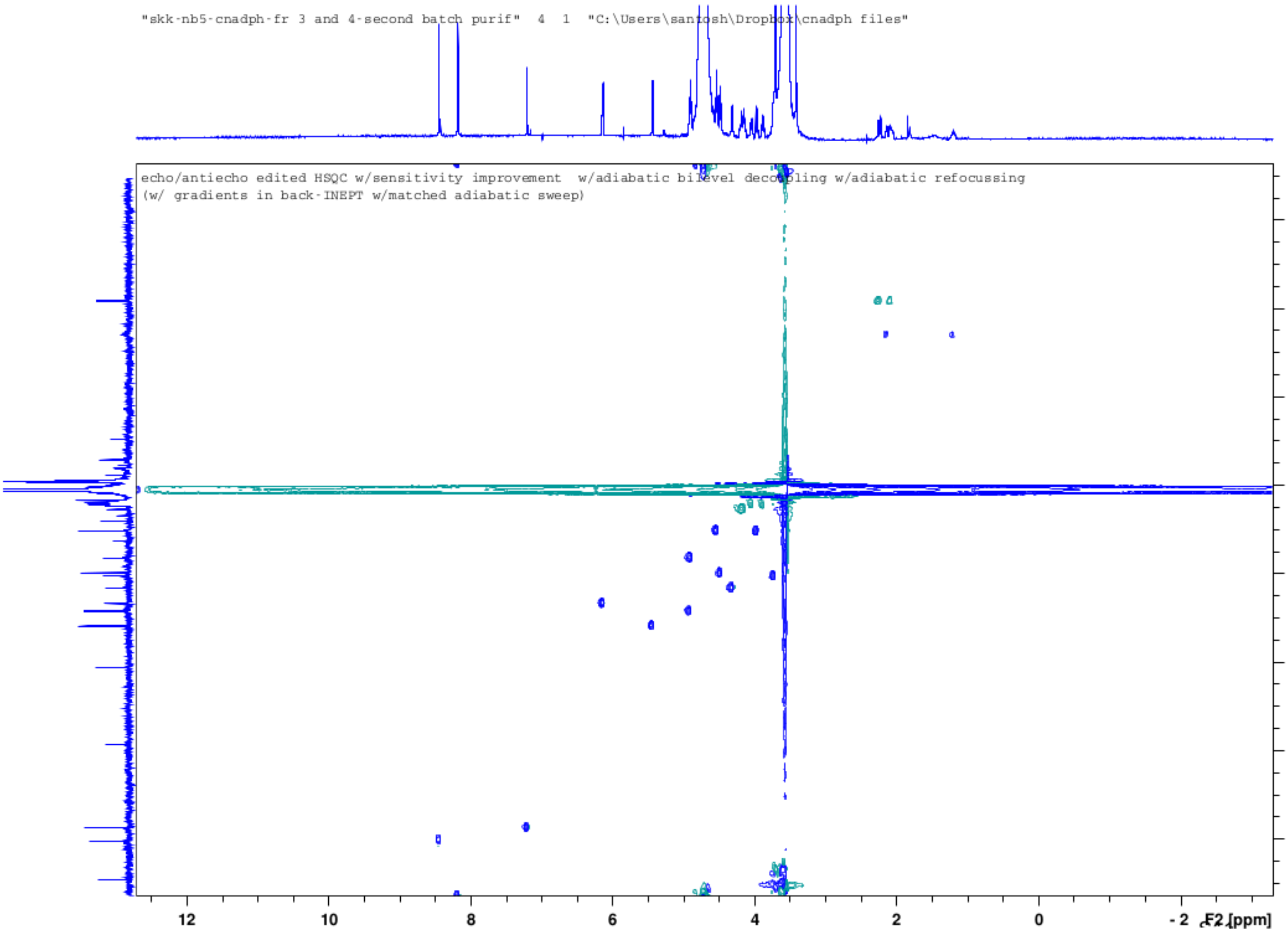
"skk-nb5-cnadph-fr 3 and 4-second batch purif" 4 1 "C:\Users\santosh\Dropbox\cnadph files"
echo/antiecho edited HSQC w/sensitivity improvement w/adiabatic bilevel decoupling w/adiabatic refocussing
(w/ gradients in back-INEPT w/matched adiabatic sweep)
F1 [ppm]
20
40
60
80
100
120
140
12
10
8
6
4
2
0
-2
F2 [ppm]

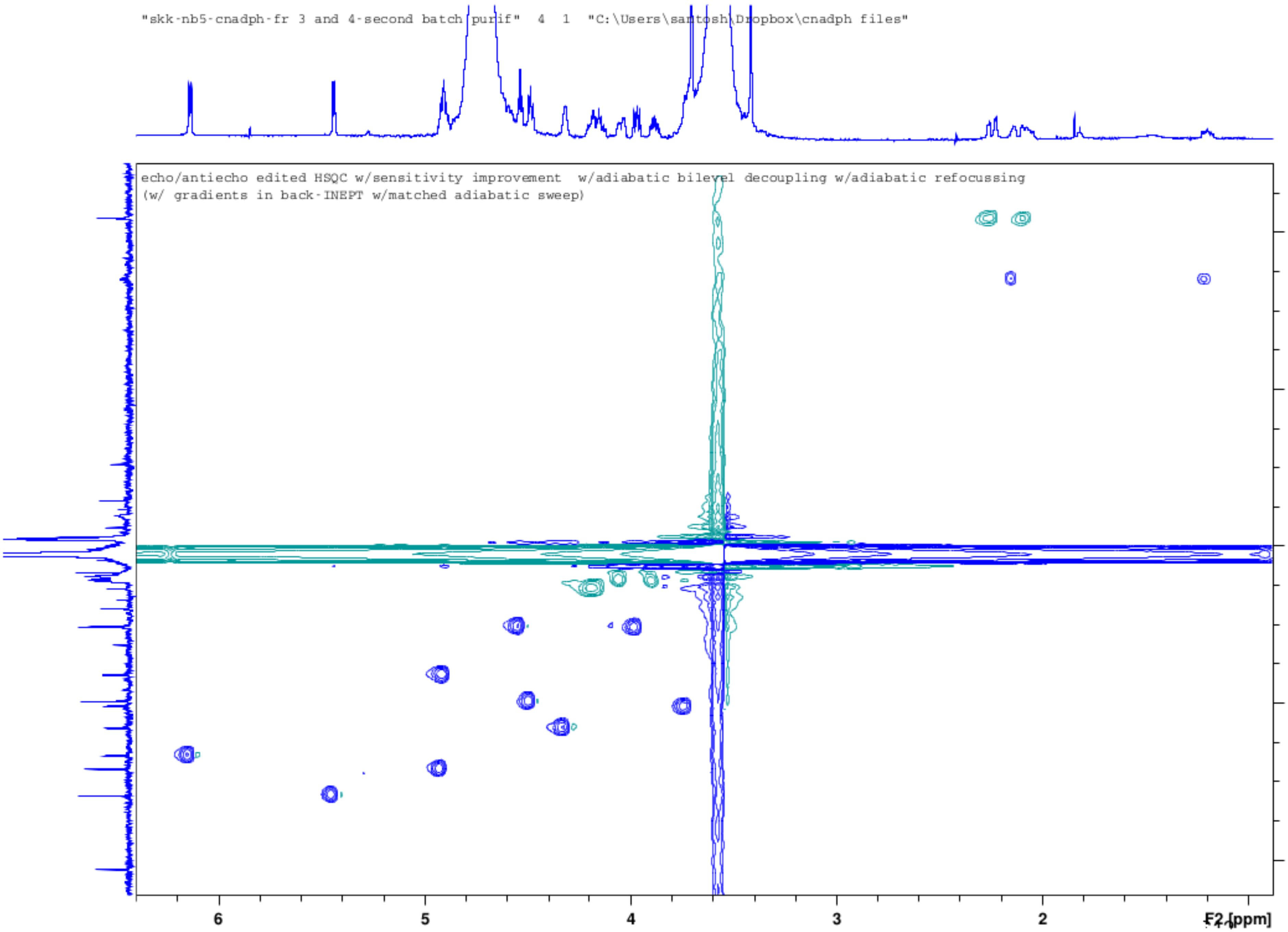

"skk-nb5-cnadph-fr 3 and 4-second batch purif" 4 1 "C:\Users\santosh\Dropbox\cnadph files"
echo/antiecho edited HSQC w/sensitivity improvement w/adiabatic bilevel decoupling w/adiabatic refocussing
(w/ gradients in back-INEPT w/matched adiabatic sweep)
F1 [ppm]
40
60
80
100
6
5
4
3
2
F2 [ppm]
