# Supplementary material for: Chiral evasion and stereospecific antifolate resistance in Staphylococcus aureus
Source: PLoS Comput Biol. 2022 Feb 10;18(2):e1009855. doi: 10.1371/journal.pcbi.1009855 (PMC8865654; doi:10.1371/journal.pcbi.1009855)
Supplement: Attachment — Submitted filename: PLOSCB_review.pdf [file pcbi.1009855.s010.pdf]

The authors findings, if true, would be a highly significant contribution to the field. The identification of an oxidoreductase enzyme that in the presence of an inhibitor binds and utilizes α-NADPH rather than β-NADPH would be unprecedented. Unfortunately, the experimental evidence for this hypothesis is marginal at best and simpler explanations exist for the difference in the density maps in the ternary complexes of S-27:NADPH:SaDHFR and R-27:NADPH:SaDHFR. For instance, binding of R-27 might perturb the interactions of the cofactor with the enzyme resulting in a broader structural ensemble of bound β-NADH conformations, producing density maps which no single conformation of β-NADH can satisfy. Even if the assignment of t-NADPH in the crystal structure of the R-27:NADPH:SaDHFR complex is correct, the relevant citations supporting the formation of tricyclic acid degradation products of the nicotinamide cofactors (#45 and #46 in the paper) both state in their abstracts that the acid product of NADPH degradation is *always* the α anomer regardless of the configuration of the starting compound (α or β). These citations both note that epimerization to the α anomer configuration is the first step in the formation of t-NADPH. ***Therefore, the presence of t-NADPH in these crystal structures would not support the unprecedented conclusion that α-NADPH is being recruited to the enzyme, since β-NADPH, according to the cited references, would also be expected to yield t-NADPH.***

Additionally, citations employed by the authors to support the binding of alternative forms of NADPH to DHFR (citations #41-44) only pertain to the *conformation* of NADPH, and not the *configuration* of it's anomeric carbon and so do not provide precedence for different configurations of NADPH binding to DHFR or any other enzyme. The author's conclusion that the NADPH cofactor of the R-27:NADPH:SaDHFR ternary complex is in the α form is therefore only supported by their OSPERY based computational analysis. As they note in the below quoted text from the paper, no structure deposited in the PDB with electron density that could accommodate either α-NADPH or t-NADPH, including their own, has sufficient resolution to conclusively assign the configuration of the anomeric carbon of NADPH.

"We searched for similar geometry among over 1700 PDB structures, but only very few were found and among them none was identified that had high enough resolution to conclude anything regarding their anomeric configuration."

Given the exceptional nature of the claims, some other form of experimental evidence of α-NADH binding the SaDHFR is warranted. The ability of their modeling methodology to evaluate the binding of α-NADH to the enzyme *in silico* is insufficient evidence to support the conclusions of the paper since the phenomenon of chiral specificity is pervasive throughout the fields of enzymology and protein-ligand interaction analysis.

Based on the differences in the density map of the ternary complexes and the proposed recruitment of α-NADPH to the active site by the R-27 compound, the authors suggest a new phenomenon they call "chiral evasion" wherein "an enzyme exploits the configuration and chirality difference of its cofactor to evade an inhibitor." Identification of such a phenomenon, if it were well supported, would be highly significant. Unfortunately, lacking any firm evidence that α-NADPH is actually recruited either *in vivo* or *in vitro* other than the assignment of t-NADPH in the crystal structure of the R-27:NADPH:SaDHFR ternary complex, the supposition that such a phenomenon exists is tenuous at best. The observed difference in efficacy of the enantiomers of compound 27 is not in and of itself unusual, and does not require any novel mechanism to explain since Protein-ligand interactions are routinely found to be dependent on the chirality of the ligand since the protein molecules are themselves chiral.

Problematically, the citations provided for the formation of t-NADPH all involve acid induced cyclization *in vitro*, which the authors propose may occur in their crystal structure owing to the acidity of the crystallization condition, while later in the paper they suggest that

"These results indicate that in contrast to S-27, which competes with DHF to bind DHFR:β-NADPH, the mechanism of inhibition of R-27 may come from its ability to bind and trap SaDHFR with the inactive t-NADPH."

It is not clear how R-27 could act in this way when t-NADPH is not present in cells. Their computational studies conclude that "In both WT or F98Y DHFR, R-27 is predicted to bind to t-NADPH:DHFR with higher affinity than to β-NADPH:DHFR (Table 2)" this result is interesting but not convincing since there is no experimental evidence that t-NADPH can form *in vivo.*

In summary, while the manuscript is well written and the computational modeling of the proposed ternary complexes well done, the absence of sufficient experimental evidence to support the extraordinary claim of recruitment of α-NADPH to the enzyme makes the computational aspects of the work highly speculative. It is highly problematic that both of the critical references for the acid induced cyclization of NADPH to t-NADPH state that the configuration of the starting material has no impact on the configuration of the t-NADPH product. Observation of t-NADPH in the crystal structure would therefore not support the conclusion that SaDHFR is in fact binding α-NADPH in the presence of R-27. Revision of the manuscript to include convincing experimental evidence of α-NADH binding would provide the necessary foundation to support the computational studies.

Recommendation: Publish with major revisions, in particular, with strong experimental evidence that the phenomenon of cooperative recruitment of α-NADPH in the presence of R-27 actually occurs. If such evidence could be provided then the paper would report findings that would be, in my opinion, highly significant to the field.
