# Supplementary material for: Chiral evasion and stereospecific antifolate resistance in Staphylococcus aureus
Source: PLoS Comput Biol. 2022 Feb 10;18(2):e1009855. doi: 10.1371/journal.pcbi.1009855 (PMC8865654; doi:10.1371/journal.pcbi.1009855)
Supplement: Attachment — Submitted filename: rtr.pdf [file pcbi.1009855.s012.pdf]

How we responded to the reviewers' comments: Chiral evasion and stereospecific antifolate resistance in Staphylococcus aureus

Siyu Wang   Stephanie M. Reeve   Graham T. Holt
Adegoke A. Ojewole   Marcel S. Frenkel   Pablo Gainza
Santosh Keshipeddy   Vance G. Fowler   Dennis L. Wright
Bruce R. Donald

We thank the reviewers for their suggestions on our manuscript. Below, we address each comment individually and summarize the resulting changes to the manuscript. We have not only clarified language in the revised manuscript but also added data from new experiments we performed, and we describe how these new data support our claims. The new data include enzyme activity assays that indicate that t-NADPH binds to SaDHFR and furthermore binds cooperatively with R-27 (See Section S3 and Figure S5), and NMR and HPLC data that indicate that populations of α-NADPH and t-NADPH are present under physiologically relevant conditions (See Table S3, Figures S1, S3, and S4, and Section S6).

While changes to the manuscript or SI have been made throughout, the major ones are indicated in red. References can be found at the end of this response. We note that reference numbers provided within this response refer to the internal response bibliography and not to the manuscript bibliography.

Reviewer 1

> The authors' findings, if true, would be a highly significant contribution to the field. The identification of an oxidoreductase enzyme that in the presence of an inhibitor binds and utilizes α-NADPH rather than β-NADPH would be unprecedented.

Answer 1: Although binding of a non-β form of NADPH to DHFR enzymes is not precisely unprecedented [4], it does appear to be rare. However, we thank the reviewer for their positive estimation of the impact of our work. We clarify our arguments and provide new data in response to the reviewer's comments that we believe support our findings.

> Unfortunately, the experimental evidence for this hypothesis is marginal at best ...

Answer 2: The reviewer suggests that existing experimental data is not sufficient to discard the possibility that the NADPH cofactor in these structures is the β anomer. In this answer we present the crystallographic evidence supporting our claim that, in the structure of R27:NADPH:SaDHFR, the NADPH cofactor cannot be the β form and is in fact t-NADPH. In response to the reviewer's later comments we are pleased to add new experimental data (See Answer 6) supporting this claim to the revised manuscript. We thank the reviewer for noting that our presentation of the crystallographic evidence was unclear, and in response to their comments we have updated the revised manuscript.

It has been shown conclusively for several structures of SaDHFR complexed with inhibitors and NADPH, including some structures treated in this paper, that the cofactor geometry precludes assigment of the β-epimer [2, 4]. For example, the 1.77 Å resolution structure of SaDHFR (F98Y) with the inhibitor UCP115A (PDB ID: 3FQF) [1] provided extremely well-resolved density around the ribose sugar of the NADPH cofactor. Detailed analysis [2] revealed that the assignment of the β-anomer to this density would require that the bond geometry of the anomeric carbon deviate from ideal tetrahedral geometry by 75°, indicating that the cofactor cannot be the β-anomer — it must be α- or t-NADPH. Indeed, assigning α-NADPH to this density resulted in much more regular bond geometry. The structures treated here in the current manuscript were solved later [4], and the structure of R-27:NADPH:SaDHFR was assigned the α-NADPH cofactor after similar analysis. In sum, there exist several published, high-resolution crystal structures for which no single conformation of β-NADPH that respects typical bond geometries can be fit to the cofactor density. We have updated the text in several places to clarify the crystallographic evidence that precludes the assignment of β-NADPH:

> To investigate more closely we examined the density map of 3fqf, 3fqo, 3fqv, 3fqz, and measured the geometry (i.e., bond angles) of NADPH around the chiral center (C1′ of ribose on nicotinamide side) [2]. The electron density around the ribose sugar of NADPH is extremely well-resolved for both 3fqf and 3fqz, and the best-fit β-NADPH conformation deviated by up to 75° from ideal tetrahedral geometry at the anomeric center. This analysis showed conclusively that the epimer in these structures is *not* β-NADPH.

Furthermore, in the revised manuscript we show that crystallographic evidence strongly supports the assignment of t-NADPH over α-NADPH. The remediated protein structure to SaDHFR with S-27 bound to t-NADPH better satisfies the experimental crystallography data assessed both locally within the cofactor binding site and globally using statistical metrics for the entire crystal structure. Locally, and perhaps most importantly, t-NADPH satisfies the difference density that was present with α-NADPH. Additionally, the assigment of t-NADPH results in a decrease in the average B-factors (from 39.98 with α-NADPH to 33.55 with t-NADPH) and the B-factors of the oxygen atom involved in cyclization (from 39.22 for α-NADPH to 34.15 with t-NADPH). Globally, the decrease in the overall R-free factors is significant given the small change in the overall structure. Combined, this evidence suggests that t-NADPH is the predominant cofactor in the NADPH binding site. To clarify the crystallographic evidence that supports the assignment of t-NADPH over α-NADPH we have made the following changes to the manuscript on page 13, paragraph 2:

Upon refinement of these two structures in PHENIX, we observed that the tNADPH cofactor better satisfied the local electron density map and difference density that arose in the α-NADPH structure. Additionally, we observe a decrease in the average cofactor B-factors of 39.98 with a-NADPH and 33.55 with tNADPH as well as the B-factors of the oxygen atom involved in cyclization with a reduction from 39.22 for a-NADPH to 34.15 with tNADPH. Furthermore, there is an overall decrease in Rfree for the structure with the remediation of the NADPH cofactor, with a decrease from 0.2543 with a-NADPH to 0.2529 with tNADPH, a meaningful decrease given the minor structural differences between cofactor structures.

. . . and simpler explanations exist for the difference in the density maps in the ternary complexes of S-27:NADPH:SaDHFR and R-27:NADPH:SaDHFR. For instance, binding of R-27 might perturb the interactions of the cofactor with the enzyme resulting in a broader structural ensemble of bound β-NADH conformations, producing density maps which no single conformation of β-NADH can satisfy.

Answer 3: The reviewer proposes an interesting alternative hypothesis to explain the cofactor electron density. However, we believe that their hypothesis is both more complicated and less consistent with crystallographic data.

The reviewer proposes that a broad structural ensemble of bound β-NADPH conformations might satisfy the density better than either α-NADPH or t-NADPH. We have found no evidence for the existence of such an ensemble. In particular, the density around the NADPH cofactor binding site is well-resolved, providing evidence counter to the claim that there exists a broad ensemble of NADPH conformations in these crystals. It would be highly speculative and violate the principle of parsimony (Occam's razor) to assign such a complex model when the density is well-satisfied by the assignment of a single conformation of t-NADPH – a species that is present in solution under crystallographic conditions as evidenced by NMR and HPLC data (Table S3). We have added these new data to the revised manuscript to address the reviewer's concerns and support the presence of t-NADPH. Additionally, we have explicitly acknowledged the alternative hypothesis and our reasons for discarding it in the Discussion section:

We also considered the possibility that the density results from the averaging of a broad ensemble of β-NADPH conformations, but discarded this hypothesis due to its complexity and the well-resolved density around the cofactor.

Even if the assignment of t-NADPH in the crystal structure of the R-27:NADPH:SaDHFR complex is correct, the relevant citations supporting the formation of tricyclic acid degradation products of the nicotinamide cofactors (#45 and #46 in the paper) both state in their abstracts that the acid product of NADPH degradation is always the α anomer regardless of the configuration of the starting compound (α or β). These

citations both note that epimerization to the α anomer configuration is the first step in the formation of t-NADPH. Therefore, the presence of t-NADPH in these crystal structures would not support the unprecedented conclusion that α-NADPH is being recruited to the enzyme, since β-NADPH, according to the cited references, would also be expected to yield t-NADPH.

Answer 4: We agree with the reviewer that our hypothesis – that α-NADPH is recruited to the active site and undergoes ring-closure *in situ* – is not the only hypothesis supported by our crystal structure. We do recognize that binding of R-27 to DHFR: β-NADPH binary complex could result in both anomerization and subsequent ring closure *in situ*. However, given that α-NADPH is a biochemically and physiologically relevant species in solution (See Answer 6 below, Figure S5, Table S3), a model that requires both anomerization and ring closure to occur in the enzyme active site (henceforth: the alternative hypothesis) is more complicated than the model proposed in the original manuscript that merely requires ring closure *in situ*. Hence, we give the alternative hypothesis less weight. Regardless of the precise step-wise mechanism this enzyme exhibits different, enantiomerically-induced preferences for tertiary complexes, which is the main focus of our argument.

We thank the reviewer for pointing out that language in the manuscript could be interpreted as endorsing only one step-wise binding mechanism. We now acknowledge the alternative binding hypotheses and also explicitly acknowledge future work to probe the kinetics of this system in the revised manuscript:

> Although the crystallographic and *in vitro* evidence supporting the relevance of t-NADPH to this system is clear, the role of t-NADPH in cells is unknown. We propose several possibilities: Our examination of populations of α-NADPH and t-NADPH by HPLC and NMR under various conditions indicate that trace amounts of t-NADPH can be observed under physiologically-relevant conditions (Table S3). Alternatively, given the structural similarity between α-NADPH and t-NADPH, it is plausible that α-NADPH could fill the role of t-NADPH in this mechanism. Due to the rapid interconversion between α-NADPH and β-NADPH we could not perform binding experiments with pure samples of α-NADPH. It is also possible that anomerization or cyclization could occur within the enzyme *in situ* in solution, or perhaps only under crystallographic conditions. However, due to the experimental evidence herein supporting t-NADPH binding to SaDHFR (Section S3, Figure S5), we believe this last possibility to be less likely. Further study will be required to conclusively assign the role of t-NADPH *in vivo*. Finally, previous work has suggested that the F98Y mutation may affect binding kinetics [4]. The computational modeling techniques presented here provide thermodynamic information, and alternative approaches will be required to probe the kinetics of this system.

Additionally, citations employed by the authors to support the binding of alternative forms of NADPH to DHFR (citations #41-44) only pertain to the conformation of NADPH, and not the configuration of it's anomeric carbon and so do

not provide precedence for different configurations of NADPH binding to DHFR or any other enzyme. The author's conclusion that the NADPH cofactor of the R-27:NADPH:SaDHFR ternary complex is in the α form is therefore only supported by their OSPERY based computational analysis. As they note in the below quoted text from the paper, no structure deposited in the PDB with electron density that could accommodate either α-NADPH or t-NADPH, including their own, has sufficient resolution to conclusively assign the configuration of the anomeric carbon of NADPH.

> We searched for similar geometry among over 1700 PDB structures, but only very few were found and among them none was identified that had high enough resolution to conclude anything regarding their anomeric configuration.

Answer 5: To our knowledge the presence of a non-β-NADPH cofactor configuration in crystals is unique to our SaDHFR:antifolate system and has been identified three times in our structures. We have been able to conclude with certainty that the NADPH cofactor is not the β epimer in three instances (PDB IDs: 3FQF and 3FQZ [2,4], 4XEC [4]). For a detailed discussion of the crystallographic evidence supporting this claim, please see Answer 2 above. Not only do we have clear density for the NADPH in these crystal structures, but chemically, the cis-ring closure facilitated by the α-configuration is more energetically favorable and probable than the β form, supporting our assignment of the α or t-configuration.

In fact the referenced 1700 PDB structures *did not* include our own structures for the reason that our structures do allow conclusive assignment of the anomeric carbon, whereas previous structures do not. We recognize that our wording in the above quoted text could be misinterpreted as contradictory to our claims about the existence of this non-β-NADPH configuration. We have revised the text to clarify this point and address the reviewer's concerns:

> The OSPREY-based analysis [2] revealed that the alternative conformation of NADPH reported in Ref. 1 actually possesses a different configuration relative to β-NADPH at the anomeric center. To investigate more closely we examined the density map of 3fqf, 3fqo, 3fqv, 3fqz, and measured the geometry (i.e., bond angles) of NADPH around the chiral center (C1′ of ribose on nicotinamide side) [2]. The electron density around the ribose sugar of NADPH is extremely well-resolved for both 3fqf and 3fqz, and the best-fit β-NADPH conformation deviated by up to 75° from ideal tetrahedral geometry at the anomeric center. This analysis showed conclusively that the epimer in these structures is *not* β-NADPH. To determine the prevalence of this phenomenon we searched over 1700 NADPH-containing PDB structures for similar geometries at the anomeric center. At that time only 23 structures with similar NADPH geometry were found and among them only 3fqf and 3fqz had high enough resolution to conclude anything regarding their anomeric configuration.

Given the exceptional nature of the claims, some other form of experimental evidence of α-NADH binding the SaDHFR is warranted. The ability of their modeling methodology to evaluate the binding of α-NADH to the enzyme in silico is insufficient evidence to support the conclusions of the paper since the phenomenon of chiral specificity is pervasive throughout the fields of enzymology and protein-ligand interaction analysis.

Answer 6: Because α-NADPH is not commercially available and due to its interconversion to β-NADPH we are unable to perform binding experiments with pure samples of α-NADPH. However, in response to the reviewer's comments we have performed new biochemical experiments with our synthetically-isolated t-NADPH and added these data to the revised manuscript (Table S3). Due to the strong structural similarity between t-NADPH and α-NADPH we believe that it is reasonable to use t-NADPH as a proxy for α-NADPH.

In response to the reviewer's comments we provide additional experimental data that support the claim that t-NADPH (α-NADPH by proxy) can bind to SaDHFR. First, we have determined that t-NADPH is not a substrate of SaDHFR (See Section S3.3). Second, we have determined that t-NADPH binds to SaDHFR through our enzyme kinetics experiments: t-NADPH is a poor inhibitor of SaDHFR with an IC_{50} of 111 ± 6 μM. Third, co-incubation of β-NADPH and t-NADPH (1:1) with R-27 led to a 2-fold reduction in R-27 IC_{50} (See Figure S5, additions to the SI), suggesting co-operativity of binding between t-NADPH and R-27. Together, these new data indicate that t-NADPH or α-NADPH are able to bind to SaDHFR. Finally, we performed experiments to assess the populations of α-NADPH and t-NADPH under various conditions, demonstrating that populations of α-NADPH and t-NADPH are present under physiologically relevant conditions (Table S3). Data for these requested experiments have now been added to the supplemental material and are reflected in the revised manuscript.

To address the reviewer's request we have added Figure S5 and Table S3 to the SI. We have added method descriptions for these new experiments in Section S3 of the SI. Additionally, we have revised the main manuscript on page 13-14 to describe these additional experiments:

> β-NADPH and α-NADPH are both physiologically relevant species [7]. They naturally interconvert although the β form is more stable and generally the isomer utilized by DHFR. We have observed via HPLC analysis that α-NADPH accounts for 7% of the concentration of NADPH at neutral pH (Table S3). However at low pH α-NADPH can become trapped as t-NADPH by undergoing a cyclization through the addition of the 20 hydroxyl on the ribose 13 and the tetrahydronicotinamide ring [5, 6]. We also note that the acid treatment of β-NADPH also leads to t-NADPH via initial anomerization to the α form [6]. To understand the prevalence of α-NADPH and subsequent formation of t-NADPH, we used NMR to track the species in biochemical, microbiological and crystallographic conditions (Table S3). Similar to published work [5, 6], under acidic conditions (pH 4.5, RT) we see enrichment of α-NADPH to 14% of the total solution and after 7h were able to isolate 30% t-NADPH. Having isolated t-NADPH, we were able to perform biochemical experiments with SaDHFR [4]. We found that alone,

t-NADPH is not an active cofactor for SaDHFR. Additionally, we were able to identify that t-NADPH alone has inhibitory effects on SaDHFR with an IC_{50} of 111 ± 6 μM. Additionally, we have shown that co-incubation of t-NADPH with R-27 and SaDHFR and enzymatic activation with a mix of β-NADPH and DHF results in a 2-fold reduction in IC_{50} compared to no t-NADPH pre-incubation (Figure S5).

Based on the differences in the density map of the ternary complexes and the proposed recruitment of α-NADPH to the active site by the R-27 compound, the authors suggest a new phenomenon they call "chiral evasion" wherein "an enzyme exploits the configuration and chirality difference of its cofactor to evade an inhibitor." Identification of such a phenomenon, if it were well supported, would be highly significant. Unfortunately, lacking any firm evidence that α-NADPH is actually recruited either *in vivo* or *in vitro* other than the assignment of t-NADPH in the crystal structure of the R-27:NADPH:SaDHFR ternary complex, the supposition that such a phenomenon exists is tenuous at best.

Answer 7: Please refer to Answers 2 and 6 above. Briefly, in addition to crystallographic evidence supporting the presence of t-NADPH in the crystal structure of the R-27:NADPH:SaDHFR ternary complex, we have added in the revised manuscript *in vitro* experiments demonstrating that t-NADPH is a weak inhibitor of SaDHFR and supporting cooperativity of binding between t-NADPH and R-27. These experiments provide additional support for the relevance of an alternative form of NADPH.

The observed difference in efficacy of the enantiomers of compound 27 is not in and of itself unusual, and does not require any novel mechanism to explain since Protein-ligand interactions are routinely found to be dependent on the chirality of the ligand since the protein molecules are themselves chiral.

Answer 8: It is indeed common for protein-ligand interactions to be dependent on ligand chirality. Such ligands can be important probes for understanding ligand binding. This manuscript discusses a specific system in which differential efficacy of the enantiomers of compound 27 is induced by the F98Y mutation in SaDHFR. To be clear – without the F98Y mutation, R-27 and S-27 inhibit SaDHFR with approximately equal potency. However, the mutation F98Y causes R-27 to become a significantly less potent inhibitor of SaDHFR (F98Y) than S-27. To our knowledge the induction of stereoselectivity by a single, conservative mutation is unusual, if not unique.

Furthermore, crystal structures of SaDHFR (WT) bound to enantiomerically pure isomers of compound 27 (PDB ID: 4TU5, 7T7S) show a unique active site plasticity that allows the enzyme to display preferences for different configurations for the cofactor NADPH. These structures, supported by biochemical data, also show that the enzyme is able to accommodate both isomers with high affinity by the formation of a cryptic inhibitor binding site.

These two observations together demonstrate an unusual and significant biological phenomenon that we feel warrants the development of a model to understand the thermodynamic principles that contribute to these chiral preferences.

Of additional importance is the fact that perturbation of the NADPH and its effects on cooperative binding are known contributors to clinical trimethoprim resistance [3] and we believe that our work to develop a model of F98Y-mediated resistance and understand the chiral preferences of DHFR is of utmost importance as we continue to investigate TMP resistance and develop new inhibitors against this target.

> Problematically, the citations provided for the formation of t-NADPH all involve acid induced cyclization *in vitro*, which the authors propose may occur in their crystal structure owing to the acidity of the crystallization condition, while later in the paper they suggest that "These results indicate that in contrast to S-27, which competes with DHF to bind DHFR:β-NADPH, the mechanism of inhibition of R-27 may come from its ability to bind and trap SaDHFR with the inactive t-NADPH."

Answer 9: The reviewer is correct that we acknowledge that our crystallographic conditions can cause the enrichment of t-NADPH in solution due to incubation at an acidic pH. Following this statement in the original manuscript, we also suggested that the active site could lower the pKa of the NADPH and facilitate the cyclization *in situ*. These statements mentioned above are merely intended to acknowledge a few of the possible hypotheses regarding the role of α- or t-NADPH in solution for binding with R-27 in DHFR, as the biological role of these species is not yet clear. We thank the reviewer for pointing out that these statements could be interpreted as contradictory, and have revised the manuscript to more clearly acknowledge the various hypotheses for the *in vivo* sources and roles of α-NADPH and t-NADPH:

> Although the crystallographic and *in vitro* evidence supporting the relevance of t-NADPH to this system is clear, the role of t-NADPH in cells is unknown. We propose several possibilities: Our examination of populations of α-NADPH and t-NADPH by HPLC and NMR under various conditions indicate that trace amounts of t-NADPH can be observed under physiologically-relevant conditions (Table S3). Alternatively, given the structural similarity between α-NADPH and t-NADPH, it is plausible that α-NADPH could fill the role of t-NADPH in this mechanism. Due to the rapid interconversion between α-NADPH and β-NADPH we could not perform binding experiments with pure samples of α-NADPH. It is also possible that anomerization or cyclization could occur within the enzyme *in situ* in solution, or perhaps only under crystallographic conditions. However, due to the experimental evidence herein supporting t-NADPH binding to SaDHFR (Section S3, Figure S5), we believe this last possibility to be less likely. Further study will be required to conclusively assign the role of t-NADPH *in vivo*.

Please refer to Answers 6 and 10 for a more detailed discussion of the question of *in vivo* relevance of t-NADPH and α-NADPH.

It is not clear how R-27 could act in this way when t-NADPH is not present in cells. Their computational studies conclude that "In both WT or F98Y DHFR, R-27 is predicted to bind to t-NADPH:DHFR with higher affinity than to β-NADPH:DHFR (Table 2)" this result is interesting but not convincing since there is no experimental evidence that t-NADPH can form *in vivo*.

Answer 10: We agree with this reviewer that the biological prevalence of t-NADPH is not known. However, it is known that the α-NADPH precursor is a biologically relevant species [7] and is present under physiological conditions (Table S3). We propose several possibilities. First, the structural similarity between t-NADPH and α-NADPH suggests that α-NADPH could perform the proposed role of t-NADPH *in vivo*. As stated above, the transience of α-NADPH and the relative stability of t-NADPH make t-NADPH a much more viable probe for *in vitro* experiments and may account for the presence of t-NADPH in these crystals. Second, as we suggest in the text, it is possible that the active site could facilitate *in situ* cyclization of α-NADPH.

Understanding the microbiological significance of this phenomenon and the role it may play in mutational antifolate resistance is of great importance as we continue to develop this class of antifolates. However, addressing the specific question of the *in vivo* relevance of t-NADPH will require an approach that we believe is beyond the scope of this manuscript. We have made sure to clarify this in the text. Please see Answers 4 and 9 which describe the relevant changes to the manuscript.

In summary, while the manuscript is well written and the computational modeling of the proposed ternary complexes well done, the absence of sufficient experimental evidence to support the extraordinary claim of recruitment of α-NADPH to the enzyme makes the computational aspects of the work highly speculative. It is highly problematic that both of the critical references for the acid induced cyclization of NADPH to t-NADPH state that the configuration of the starting material has no impact on the configuration of the t-NADPH product. Observation of t-NADPH in the crystal structure would therefore not support the conclusion that SaDHFR is in fact binding α-NADPH in the presence of R-27. Revision of the manuscript to include convincing experimental evidence of α-NADH binding would provide the necessary foundation to support the computational studies.

Recommendation: Publish with major revisions, in particular, with strong experimental evidence that the phenomenon of cooperative recruitment of α-NADPH in the presence of R-27 actually occurs. If such evidence could be provided then the paper would report findings that would be, in my opinion, highly significant to the field.

Answer 11: We thank the reviewer for appreciating the computational modeling. We believe that this modeling offers an explanatory thermodynamic model to illuminate our data on chirality preference induced by a point mutation, binding site plasticity, and the structural basis of specificity. We have added new data from new experiments and made new and revised explanations to address the reviewer's concerns.

Reviewer 2

In this study, authors have used computational protein design (CPD) software suite OSPREY to explore the mechanism of this stereo-specific inhibition, namely, Staphylococcus aureus dihyrofolate reductases (SaDHFR's) chiral evasion against PLAs (propargyl-linked antifolates) enantiomers. Compared to IC_{50} data, K^* scores (which predict K_a) produced by OSPREY successfully recapitulated the ranking of PLA enantiomers' afinity and the ranking of the impact the F98Y mutation would have in the interaction. Among all complexes, K^* scores for R-27:t-NADPH:SaDHFR and S-27:β-NADPH:SaDHFR are significantly higher than for all other models, which is consistent with NADPH configuration preferences observed in crystal structure (R-27 bound with t-NADPH and S-27 bound with β-NADPH, as seen in models 6wmy and 4tu5). Ensembles of conformations of F98Y SaDHFR binding to R-27 and S-27 are predicted as well in the present study. Based on structural analysis, authors have found that different binding modes between t-NADPH (which R-27 prefers) and β-NADPH (which S-27 prefers) are likely to be a key factor of chiral evasion. Authors have shown major difference between t-NADPH and β-NADPH is how they interact with Gly93 loop on SaDHFR. Such difference may lead to clashes between Gly93 and Tyr98 in F98Y mutant, and thus may influence the thermodynamic stability of SaDHFR:NADPH binary complex, ultimately modulating the inhibition potency of R-27 and S-27.

In this whole study authors have shown how the discovery of a configuration change in NADPH can elucidate a potential mechanism of drug resistance in SaDHFR. This study suggests that the cofactor stereogenicity and chiral evasion should be taken into account when designing new drugs for F98Y SaDHFR. The use of computational drug design and protein design algorithms gained new insight, informed hypotheses in biology, and made contributions to biochemistry. The data and models authors presented in this manuscript have already been useful in medicinal chemistry campaigns for F98Y resilient inhibitors, which suggest that they will have significant value for the scientific community. I believe this is significant advancement compare to previous work from the same group and I strongly recommend for its publication.

Answer 12: We thank the reviewer for their positive evaluation of our work.

Reviewer 3

In this manuscript, the authors combine structural analysis with simulations to propose an interesting antimicrobial resistance mechanism through "chiral evasion" from the F98Y mutant of SaDHFR. The manuscript is well written and presented.

Answer 13: We thank the reviewer for their positive evaluation of our work

First, I must say that I am not well versed in X-ray crystallography and so I cannot comment on or critique this aspect of the work. I do wonder though whether the reported change in the R_{free} value from 0.2543 to 0.2529 when going from α-NADPH to t-NADPH should be considered a significant improvement though?

Answer 14: R_{free} is a global metric for the crystal structure that compares the experimental electron density with the electron density that would agree with the proposed structure, therefore even a minor decrease in R_{free} due to reassignment of the NADPH is an indication that the t-NADPH better fits the experimental data than the previously annotated α-NADPH. More importantly, however, is that t-NADPH better satisfies the *local* electron density map by satisfying the difference density that arose as a consequence of assigning α-NADPH. Additionally, assignment of t-NADPH results in a decrease in B-factors both on average and for the oxygen atom that is involved in the cyclization reaction to form the fused ring of t-NADPH. For more details, please refer to the discussion of the crystallographic evidence in response to Reviewer 1 (Answer 2 above)

To clarify the crystallographic evidence that supports the assignment of t-NADPH over α-NADPH we have made the following changes to the manuscript on page 13, paragraph 2:

> Upon refinement of these two structures in PHENIX, we observed that the t-NADPH cofactor better satisfied the local electron density map and difference density that arose in the α-NADPH structure. Additionally, we observe a decrease in the average cofactor B-factors of 39.98 with α-NADPH and 33.55 with t-NADPH as well as the B-factors of the oxygen atom involved in cyclization with a reduction from 39.22 for α-NADPH to 34.15 with t-NADPH. Furthermore, there is an overall decrease in Rfree for the structure with the remediation of the NADPH cofactor, with a decrease from 0.2543 with α-NADPH to 0.2529 with t-NADPH, a meaningful decrease given the minor structural differences between cofactor structures.

I can however say that the simulations and subsequent simulation analysis look well performed. I'm confident this article would be of interest to the readership and I would like to recommend it for publication after addressing the following minor concerns:

1. The OSPREY software is publicly available yes, but the scripts used by the authors along with the preprocessed input structures could be shared to aid reproducibility.
2. It would be instructive to provide error estimates on the bar chats for Figure 3.
3. The label sizes on Figures 4-6 should be increased.
4. Can the authors comment on the variability of the results obtained for different snapshots from their ensemble with the Probe dots analysis. It would be good to confirm the lowest energy conformer can adequately represent the ensemble obtained from OSPREY. I mean in terms of the subsequent insights taken from the Probe dots calculations.

5. Some very minor corrections I spotted along the way: "It has been proved in Ref. 30 that K* will be equal to Ka under the condition of using exact" (add "be"). "Under this model, the ability of DHFR to readily form binary complexes..." (replace from with form).

Answer 15:

1. We have made all scripts and input structures publicly available from the Harvard Dataverse repository (`https://dataverse.harvard.edu/dataset.xhtml?persistentId=doi:10.7910/DVN/5AQP7Z`). We have updated the data availability statement in the revised manuscript to reflect this.

2. We have added error bars to Figure 3.

3. We have increased the label font size for Figure 4, 5, and 6.

4. For all ternary complexes the active site is extremely restricted, and as a consequence the side-chain, cofactor, and drug conformations in the vicinity of residue 98 are extremely similar. For these ternary complexes the lowest-energy member of the ensemble is sufficient to represent the 30 lowest-energy ensemble members, and the Probe-dot analysis remains the same across the ensemble.

   However, the binary complexes are less restricted, and as a result in some cases the variability around residue 98 for the 30 lowest-energy ensemble members is better-represented by two low-energy conformations. In particular, small, intra-rotamer changes in the side-chain of residue 98 are represented as a minor population within the low-energy ensemble for binary complexes of t-NADPH:SaDHFR, and t-NADPH:SaDHFR(F98Y). This variation in the side-chain of residue 98 appears concomitantly with a change in rotamer at V6, and is most marked in the ensemble of t-NADPH:SaDHFR. This ensemble-based analysis complements the original analysis of the lowest-energy member of the ensemble, suggesting that more space is available in the active site when t-NADPH is bound to SaDHFR, enabling the t-NADPH:SaDHFR binary complex to better-accomodate the F98Y mutation. However, this extra space is not available in the t-NADPH:R-27:SaDHFR complex (indicated by the relative restriction of the F98 side-chain in this ternary complex model), and as clashes induced by the F98Y in the ternary complex cannot be resolved.

   We have added sentences to the revised manuscript describing the variability (or lack thereof) in the low-energy ensembles for binary and ternary complexes:

   > Additionally, inspection of an ensemble of the 30 lowest-energy conformations for t-NADPH -related binary complexes reveals a minor population characterized by small (intra-rotamer) changes to residue 98 and a corresponding change in rotamer of residue V6 (Figure S6). The existence of this minor population suggests that the active sites of t-NADPH binary complexes may be less tightly-packed than all other modeled states (i.e. ternary states and β-NADPH binary complexes), for which no minor populations were predicted at these residues.

Additionally, we have added a figure to the SI visualizing the variabililty around residue 98 for binary complexes involving t-NADPH.

5. We have addressed the indicated typographical errors in the revised manuscript.

References

[1] Kathleen M Frey, Jieying Liu, Michael N Lombardo, David B Bolstad, Dennis L Wright, and Amy C Anderson. Crystal structures of wild-type and mutant methicillin-resistant Staphylococcus aureus dihydrofolate reductase reveal an alternate conformation of NADPH that may be linked to trimethoprim resistance. *Journal of molecular biology*, 387(5):1298–1308, 2009.

[2] Pablo Gainza. *Computational Protein Design with Ensembles, Flexibility and Mathematical Guarantees, and its Application to Drug Resistance Prediction, and Antibody Design.* PhD thesis, Duke University, 2015.

[3] Holly Heaslet, Melissa Harris, Kelly Fahnoe, Ronald Sarver, Henry Putz, Jeanne Chang, Chakrapani Subramanyam, Gabriela Barreiro, and J Richard Miller. Structural comparison of chromosomal and exogenous dihydrofolate reductase from staphylococcus aureus in complex with the potent inhibitor trimethoprim. *Proteins: Structure, Function, and Bioinformatics*, 76(3):706–717, 2009.

[4] Santosh Keshipeddy, Stephanie M Reeve, Amy C Anderson, and Dennis L Wright. Nonracemic antifolates stereoselectively recruit alternate cofactors and overcome resistance in S. aureus. *Journal of the American Chemical Society*, 137(28):8983–8990, 2015.

[5] Norman J Oppenheimer. The primary acid product of DPNH. *Biochemical and Biophysical Research Communications*, 50(3):683–690, 1973.

[6] Norman J Oppenheimer and Nathan O Kaplan. Structure of the primary acid rearrangement product of reduced nicotinamide adenine dinucleotide (NADH). *Biochemistry*, 13(23):4675–4685, 1974.

[7] Sheila L Smith and James J Burchall. α-pyridine nucleotides as substrates for a plasmid-specified dihydrofolate reductase. *Proceedings of the National Academy of Sciences*, 80(15):4619–4623, 1983.
