# Supplementary material for: Chiral evasion and stereospecific antifolate resistance in Staphylococcus aureus
Source: PLoS Comput Biol. 2022 Feb 10;18(2):e1009855. doi: 10.1371/journal.pcbi.1009855 (PMC8865654; doi:10.1371/journal.pcbi.1009855)
Supplement: Attachment — Submitted filename: Plosone_review_2nd_011722.pdf [file pcbi.1009855.s013.pdf]

The authors have made significant revisions to their manuscript. The authors now acknowledge the alternate hypothesis that *in situ* anomerization of β-NADPH followed by cyclization to t-NADPH may occur, providing the reader with an alternative explanation for the presence of t-NADPH in their crystal structures. The authors also provide some experimental evidence for the presence of a small population of t-NADPH molecules in NADPH samples under physiological conditions as well as evidence that t-NADPH binds competitively to DHFR *in vitro*. Additionally, the authors expanded analysis of the density maps improve the confidence in the assignment of t-NADPH in the eyes of this reviewer**.** By acknowledging the possibility that the putative t-NADPH observed in the crystal structures could be due to *in situ* anomerization of β-NADPH followed by cyclization to t-NADPH, the authors prepare the reader to make their own judgement between the primary hypothesis explored in the computational aspect of the work and this important alternative hypothesis. Since the authors have acknowledged this alternative hypothesis, have provided additional supporting experimental evidence for the presence of t-NADPH *in vivo*, and have provided evidence that t-NADPH can act as a competitive inhibitor of SaDHFR *in vitro*, this reviewer now feels the revised manuscript should be published for the benefit of the field.
