# Supplementary material for: Chiral evasion and stereospecific antifolate resistance in Staphylococcus aureus
Source: PLoS Comput Biol. 2022 Feb 10;18(2):e1009855. doi: 10.1371/journal.pcbi.1009855 (PMC8865654; doi:10.1371/journal.pcbi.1009855)
Supplement: Attachment — Submitted filename: Chiral evasion and stereospecific antifolate resistance in Staphylococcus aureus.docx [file pcbi.1009855.s011.docx]

**Chiral evasion and stereospecific antifolate resistance in *Staphylococcus aureus***

(PCOMBIOL-D-21-01260)

In this study, authors have used computational protein design (CPD) software suite OSPREY to explore the mechanism of this stereo-specific inhibition, namely, *Staphylococcus aureus* dihyrofolate reductases (SaDHFR's) chiral evasion against PLAs (propargyl-linked antifolates) enantiomers. Compared to IC_50_ data, K* scores (which predict K_a_) produced by OSPREY successfully recapitulated the ranking of PLA enantiomers' afinity and the ranking of the impact the F98Y mutation would have in the interaction. Among all complexes, K* scores for R-27:t-NADPH:SaDHFR and S-27:β-NADPH:SaDHFR are significantly higher than for all other models, which is consistent with NADPH configuration preferences observed in crystal structure (R-27 bound with t-NADPH and S-27 bound with β-NADPH, as seen in models 6wmy and 4tu5). Ensembles of conformations of F98Y SaDHFR binding to R-27 and S-27 are predicted as well in the present study. Based on structural analysis, authors have found that different binding modes between t-NADPH (which R-27 prefers) and β-NADPH (which S-27 prefers) are likely to be a key factor of chiral evasion. Authors have shown major difference between t-NADPH and β-NADPH is how they interact with Gly93 loop on SaDHFR. Such difference may lead to clashes between Gly93 and Tyr98 in F98Y mutant, and thus may influence the thermodynamic stability of SaDHFR:NADPH binary complex, ultimately modulating the inhibition potency of R-27 and S-27.

In this whole study authors have shown how the discovery of a configuration change in NADPH can elucidate a potential mechanism of drug resistance in SaDHFR. This study suggests that the cofactor stereogenicity and chiral evasion should be taken into account when designing new drugs for F98Y SaDHFR. The use of computational drug design and protein design algorithms gained new insight, informed hypotheses in biology, and made contributions to biochemistry. The data and models authors presented in this manuscript have already been useful in medicinal chemistry campaigns for F98Y resilient inhibitors, which suggest that they will have significant value for the scientific community. I believe this is significant advancement compare to previous work from the same group and I strongly recommend for its publication.
